# Quantitative HER2 tissue and plasma profiling predicts the activity of trastuzumab deruxtecan for breast cancer

**DOI:** 10.1038/s41698-026-01365-6

**Published:** 2026-03-13

**Authors:** Paolo Tarantino, Se-Eun Kim, Melissa E. Hughes, Ross J. Kusmick, Kalie Smith, Fara Brasó-Maristany, Nay Nwe Nyein Chan, Laia Paré Brunet, Laura Alder, Diana Garcia-Cortes, Jorge Gomez Tejeda Zanudo, Alyssa M. Pereslete, Laura Noteware, Heather Moore, Amanda E. D. Van Swearingen, Tianyu Li, Hersh Gupta, Olivia D’Amico, Alba Martini, Stefania Morganti, Jennifer Spindel, Charmaine Cook, Christine McLaughlin, Kathrin Dvir, Ana C. Garrido-Castro, Sarah Sammons, Janet Files, Kerry Sendrick, Simone Buck, Deborah Dillon, Rinath Jeselsohn, Yvonne Y. Li, Andrew D. Cherniack, Patricia LoRusso, Maryam Lustberg, Rosario Vega- León, Francisco Pardo, Justin Davis, Claudius Mueller, Brian Corgiat, Giuseppe Curigliano, Carey K. Anders, Emanuel F. Petricoin, David L. Rimm, Aleix Prat, Nabihah Tayob, Nancy U. Lin, Sara M. Tolaney

**Affiliations:** 1 Department of Medical Oncology, Dana-Farber Cancer Institute, Boston, MA USA; 2 Harvard Medical School, Boston, MA USA; 3 Department of Oncology and Hemato-Oncology, University of Milan, Milan, Italy; 4 Department of Data Science, Dana-Farber Cancer Institute, Boston, MA USA; 5 August Pi I Sunyer Biomedical Research Institute (IDIBAPS), Barcelona, Spain; 6 Reveal Genomics, Barcelona, Spain; 7 Department of Pathology, Yale University School of Medicine, New Haven, CT USA; 8 Duke Cancer Institute, Durham, NC USA; 9 Broad Institute of MIT and Harvard, Boston, MA USA; 10 Yale School of Medicine, New Haven, CT USA; 11 Vall d´Hebron Institute of Oncology (VHIO), Barcelona, Spain; 12 Ignite Proteomics, Golden, CO USA; 13 European Institute of Oncology IRCCS, Milan, Italy; 14 Center for Applied Proteomics and Molecular Medicine, George Mason University, Manassas, VA USA; 15 Clínic Barcelona Comprehensive Cancer Center, Barcelona, Spain; 16 Present Address: Yale School of Medicine, New Haven, CT USA; 17 Present Address: Reveal Genomics, Barcelona, Spain

**Keywords:** Biomarkers, Cancer, Oncology

## Abstract

Trastuzumab deruxtecan (T-DXd) is commonly used for treating metastatic breast cancer (MBC); however, traditional HER2 immunohistochemistry has largely failed to predict T-DXd activity. We reviewed survival outcomes and tested the reliability of multiple HER2 quantitative assays in predicting T-DXd’s performance among 191 patients with MBC. We demonstrate that T-DXd’s activity varies depending on the temporal evolution of HER2 immunohistochemical expression, with the longest activity observed among patients with HER2-positive disease or maintaining HER2-low disease across primary and metastatic settings. Quantitative HER2 assessment on pre-T-DXd samples showed that time-to-next treatment progressively increased by High Sensitivity-HER2 quartiles, Reverse Phase Protein Array HER2 quartiles, HER2DX ERBB2 mRNA scores and plasma-based DNADX HER2 signature tertiles (all with log-rank *p* < 0.05). Conversely, HER2 immunohistochemical subtypes showed limited predictive value for clinical outcomes. Additionally, elevated TOPO1 expression was associated with worse outcomes with T-DXd in HER2-negative breast cancer, suggesting potential relevance for payload-related markers in predicting T-DXd performance.

## Introduction

The development of novel human epidermal growth factor receptor 2 (HER2)-directed antibody-drug conjugates (ADC) has recently led to an expansion in the population responsive to anti-HER2 agents: besides their activity in HER2-positive breast cancer, meaningful activity has been observed with anti-HER2 ADCs for HER2-low, and even HER2-0 metastatic breast cancer (MBC).^1–3^ Based on this activity and the improvement of survival outcomes across multiple trials, trastuzumab deruxtecan (T-DXd) now represents an approved treatment option for nearly 90% of the patients with MBC.

Together with dramatically improving outcomes for a large population of patients, these approvals raise a multitude of questions. First, the phase 3 “DESTINY” clinical trials, which led to the approval of T-DXd in multiple indications, were conducted in large part outside the United States (US)^2,4–6^, highlighting the need to confirm the benefit and safety of the drug in US patients who may have been pretreated with different treatment regimens. Second, the approval of T-DXd for HER2-low MBC was based on a static view of this biomarker^2^, with no data available on the performance of T-DXd among patients with dynamic changes in HER2 expression over time, which have been shown to be extremely frequent.^7–10^ Third, no reliable biomarkers are available to identify patients more likely to derive long-lasting benefit from T-DXd. This represents a particularly relevant unmet need for MBC, given the current availability of three approved ADCs carrying topoisomerase 1 (TOPO1) payloads and the lack of biomarkers informing sequencing choices.^11^ In this setting, conventional immunohistochemistry (IHC) and FISH assays have proven able to distinguish patients with HER2-amplified vs. HER2 non-amplified disease, with the first subgroup demonstrating a markedly higher activity of T-DXd. However, within the large population of patients with HER2 non-amplified MBC, multiple prospective trials (e.g., J101, DESTINY-Breast04 and -06, DAISY)^2,3,12,13^ failed to demonstrate meaningful difference in T-DXd activity depending on the HER2 IHC score (0, 0 + , 1 + , 2 + ).^13^

Importantly, in recent years, several new technologies have been developed to more granularly assess HER2 in tumor samples, including proteomic, genomic, transcriptomic and plasma-based techniques.^14–19^

To evaluate the performance of novel quantitative HER2 assays in the prediction of T-DXd activity, we characterized outcomes of patients receiving T-DXd for MBC at two academic centers (Dana-Farber Cancer Institute, Duke Cancer Institute). Available tissue and/or plasma samples from these patients were profiled on novel quantitative assays (High Sensitivity-HER2 [HS-HER2^14^], a commercially available Clinical Laboratory Improvement Amendments [CLIA] Reverse Phase Protein Array-based proteomic breast cancer assay [RPPA^15^], HER2DX^16^, DNADX^17^ and OncoPanel^18,20^), exploring predictors of T-DXd activity. Additionally, to explore resistance mechanisms to T-DXd, we compared genomic alterations on circulating tumor DNA (ctDNA) collected before and after treatment with T-DXd.

## Results

We first sought to evaluate whether a dynamic assessment of semi-quantitative HER2 IHC could refine the prediction of outcomes with T-DXd besides a static use of this biomarker.

To do so, we reviewed outcomes (in terms of time-to-next treatment, TTNT, and overall survival, OS) among 191 patients treated with T-DXd, annotating the HER2 status across multiple time points (primary and metastatic tumor biopsies and/or resections). Demographic and clinicopathologic characteristics of the patients are described in Table 1. Prior to starting T-DXd, 126 patients (66.0%) had HER2-positive disease, 44 (23.0%) had HER2-low disease, and 21 (11.0%) had HER2-0 disease. Patients had received a median of four prior lines of therapy for MBC, including a median of two lines of chemotherapy. The median follow-up was 16.6 months (interquartile range [IQR]: 8.8 – 29.5).

**Table 1:** Description of the patient population by HER2 status prior to T-DXd treatment

	HER2-0 (*n* = 21)	HER2-low (*n* = 44)	HER2-positive (*n* = 126)	Total (*n* = 191)
Median age at metastatic diagnosis, years (range)	57.5 (25.4–76.5)	52.6 (27.6–70.1)	48.3 (21.4–78.0)	50.9 (21.4–78.0)
Sex				
Female	21 (100%)	44 (100%)	124 (98.4%)	189 (99.0%)
Male	0 (0%)	0 (0%)	2 (1.6%)	2 (1.0%)
Race				
African American	1 (4.8%)	2 (4.5%)	15 (11.9%)	18 (9.4%)
Asian or Pacific Islander	0 (0.0%)	0 (0.0%)	4 (3.2%)	4 (2.1%)
White	20 (95.2%)	39 (88.6%)	101 (80.2%)	160 (83.8%)
Other	0 (0.0%)	2 (4.5%)	5 (4.0%)	7 (3.7%)
Unknown	0 (0.0%)	1 (2.3%)	1 (0.8%)	2 (1.0%)
Time from metastatic diagnosis to initiation of T-DXd, months (range)	40 (11–110)	45 (2–185)	37.9 (-12–256)	40 (-12–256)
Median number of metastatic sites at diagnosis (range)	3 (1–4)	2 (1–7)	2 (0–6)	2 (0–7)
Hormone receptor status at initiation of T-DXd				
HR-positive	12 (57.1%)	30 (68.2%)	67 (53.2%)	109 (57.1%)
HR-negative	9 (42.9%)	14 (31.8%)	58 (46.0%)	81 (42.4%)
Not Done	0 (0%)	0 (0%)	1 (0.8%)	1 (0.5%)
Prior lines of treatment in the advanced setting, median (range)	4 (3–8)	4.5 (0–12)	4 (0–16)	4 (0–16)
Prior lines of chemotherapy, median (range)	2 (1–4)	2 (0–6)	2 (0–9)	2 (0–9)
Prior lines of endocrine therapy, median (range)	1 (0–4)	2 (0–5)	0 (0–5)	0 (0–5)
Treatment administered immediately after T-DXd^*^				
Chemotherapy +/- anti-HER2 mAb	5 (50%)	11 (55%)	21 (28%)	37 (35.2%)
HER2 TKI-containing regimen	0 (0%)	0 (0%)	27 (36%)	27 (25.7%)
ADC (excluding T-DM1)	3 (30%)	3 (15%)	4 (5.3%)	10 (9.5%)
T-DM1-containing regimen	0 (0%)	1 (5%)	2 (2.7%)	3 (2.9%)
Immunotherapy-based regimen	0 (0%)	2 (10%)	6 (8.0%)	8 (7.6%)
Other	2 (20%)	3 (15%)	15 (20%)	20 (19.1%)

^*^The percentages for treatments administered immediately after T-DXd are based on the number of people who received a treatment after TDXd.

HER2, human epidermal growth factor receptor 2; T-DXd, trastuzumab deruxtecan; HR, hormone receptor; TKI, Tyrosine Kinase Inhibitor; ADC, antibody-drug conjugate; T-DM1, ado-trastuzumab emtansine.

**Fig. 1:**
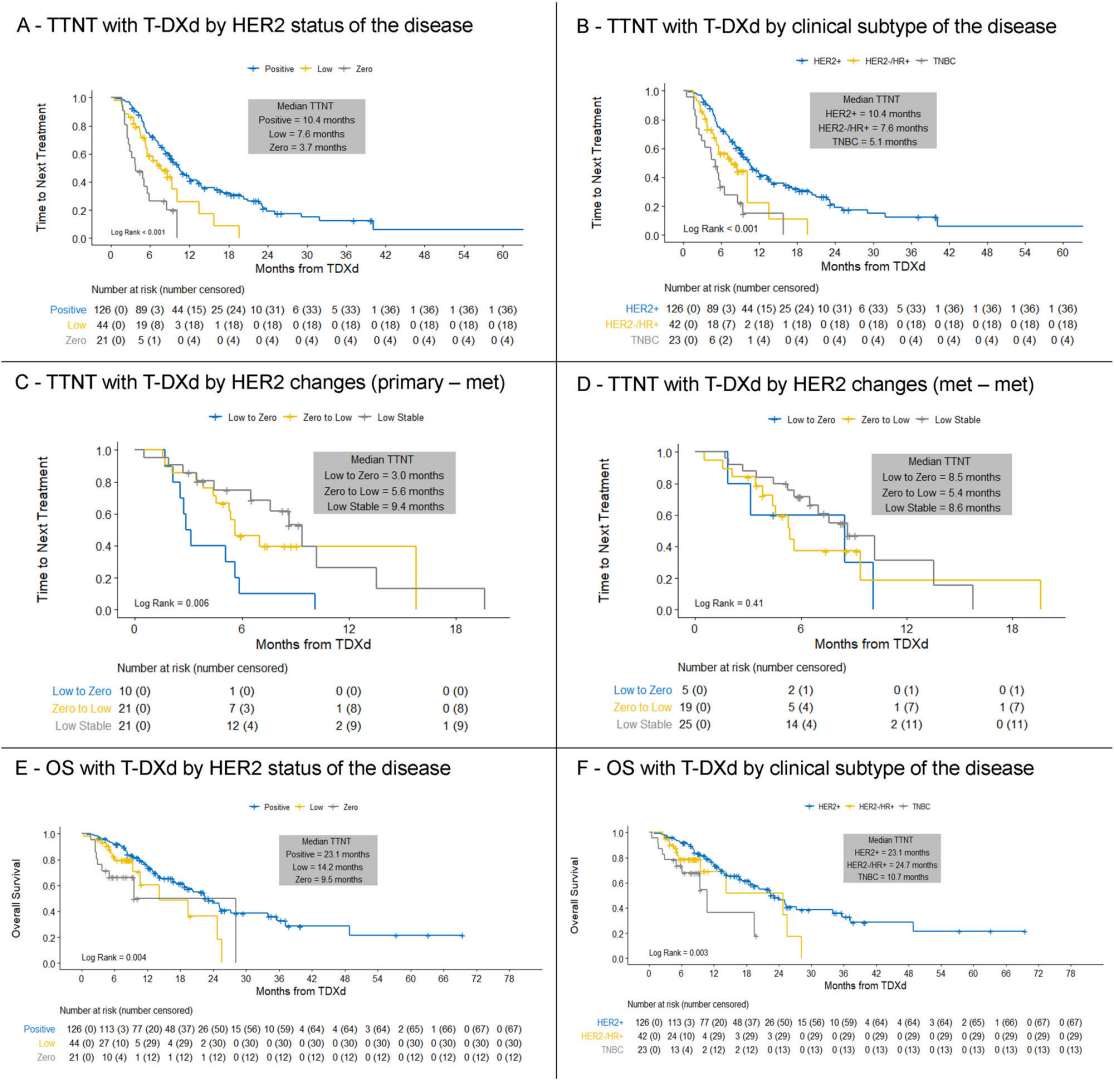
Real-world activity of T-DXd in patients with metastatic breast cancer (*n* = 191). **A** TTNT with T-DXd by HER2 status of the disease; **B** TTNT with T-DXd by clinical subtype of the disease; **C** TTNT with T-DXd depending on changes in HER2 status (low vs. 0) between the primary tumor and last metastatic biopsy; **D** TTNT with T-DXd depending on changes in HER2 status (low vs. 0) between the first and most recent metastatic biopsy; **E** OS with T-DXd by HER2 status of the disease; **F** OS with T-DXd by clinical subtype of the disease. T-DXd, trastuzumab deruxtecan; TTNT, time to next treatment; HER2, human epidermal growth factor receptor 2; OS, overall survival; HR, hormone receptor; TNBC, triple-negative breast cancer.

We initially evaluated outcomes according to static HER2 IHC and clinical subtype (HER2-positive, hormone receptor-positive/HER2-negative, triple-negative). In our cohort, TTNT and OS with T-DXd were found to vary significantly by both HER2 status (Fig. 1A; p < 0.0001) and clinical subtype (Fig. 1B; p < 0.0001).

We then focused on the subgroup of patients with HER2-low disease, where we observed extensive evolution in HER2 status (from low to 0, and vice versa). In this cohort, the temporal evolution of the HER2 status was found to be significantly associated with outcomes (Fig. 1C). TTNT was longest for patients who maintained stable HER2-low IHC status across the primary tumor and metastatic sample (9.4 months, 95% confidence interval (CI): 4.4–13.5), whereas shorter TTNT was observed for patients who switched from HER2-low to HER2-0 (3.0 months, 95% CI: 1.7–5.6) and for those who switched from HER2-0 to HER2-low (5.6 months, 95% CI: 4.4–15.8) (Fig. 1C; *p* = 0.006). Conversely, TTNT did not vary based on a change in HER2 status between two metastatic samples (Fig. 1D; *p* = 0.4119).

OS after starting T-DXd also differed significantly depending on the HER2 status (Fig. 1E; *p* = 0.0039) and clinical subtype (Fig. 1F; *p* = 0.0032). Patients with HER2-positive disease had a median OS of 23.1 months (95% CI: 18.6–27.2), followed by 14.2 months for HER2-low (95% CI: 9.4–25.5) and 9.5 months for HER2-0 MBC (95% CI: 3.7–28.2) (Fig. 1E; *p* = 0.0039). Key toxicities observed with T-DXd in this cohort are reported in Supplementary Table 1. Additionally, outcomes of treatments administered immediately after T-DXd are reported in Supplementary Figs. 1, 2. In general, outcomes with post-T-DXd regimens were relatively poor, with TTNT ranging between 4.0 months for patients with HER2-positive disease, 3.1 months for patients with HER2-low disease to 4.3 months for patients with HER2-0 disease (*p* = 0.63).

Overall, these analyses confirm that T-DXd exhibits distinct activity for different clinical subtypes of MBC and highlight a clinical relevance for the dynamic evolution of HER2 IHC score to predict the effectiveness of T-DXd in HER2-low MBC.

Importantly, while IHC HER2 status provides some prediction of T-DXd effectiveness, its limited dynamic range and subjective nature represent critical challenges in utilizing it as a biomarker for T-DXd; these challenges may have led to its lack of utility in most T-DXd phase 3 trials to date^2,3,12^. Therefore, we next sought to assess whether novel, quantitative HER2 assays would more granularly predict the effectiveness of T-DXd.

**Fig. 2:**
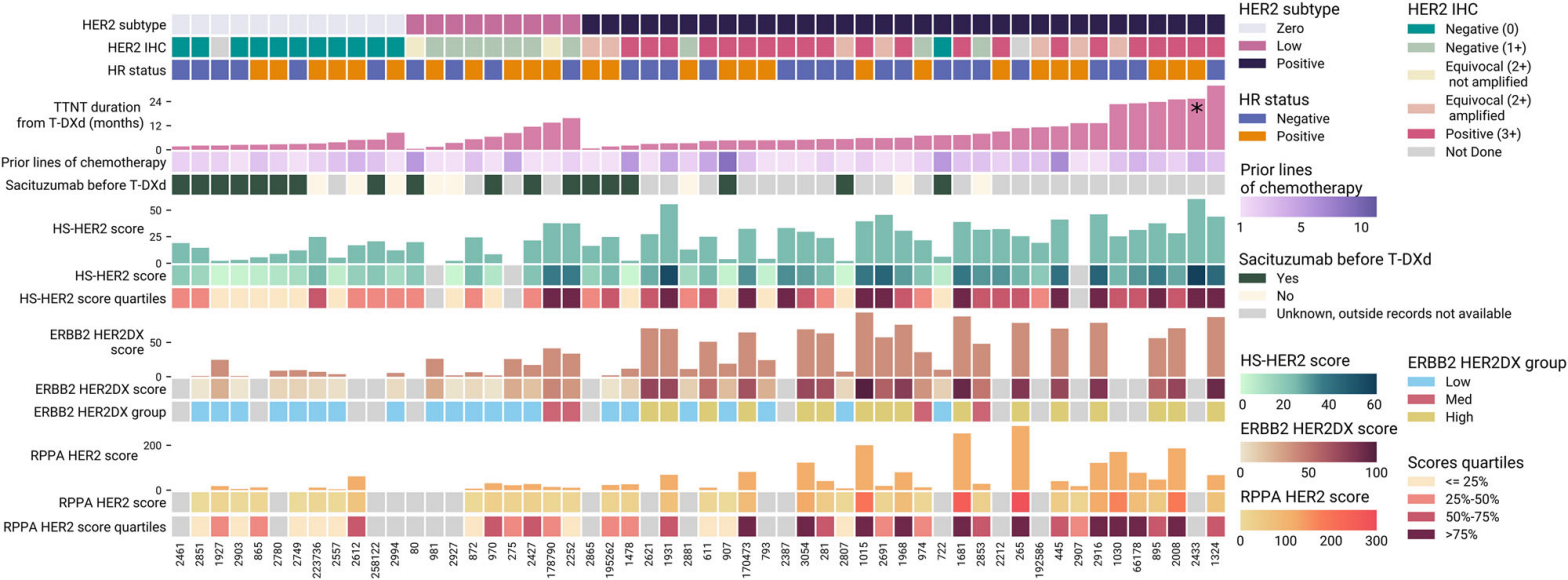
Association between time to next treatment (TTNT) for patients treated with trastuzumab deruxtecan (T-DXd) and tissue-based HER2 quantification using HS-HER2, HER2DX ERBB2, and RPPA HER2. Samples are ordered by HER2 IHC subtype and TTNT from the start of T-DXd treatment. Clinical parameters displayed include HER2 IHC subtype (HER2-zero, HER2-low, HER2-positive), latest HER2 IHC prior to T-DXd, number of prior lines of chemotherapy, and receipt of sacituzumab govitecan before T-DXd. An asterisk on the TTNT bar indicates treatment is still ongoing. Bar plots show scores for each assay, and these values are also represented with color-coded squares, along with their corresponding quartiles for the HS-HER2 and RPPA HER2 assays. For HER2DX ERBB2, score groups are displayed based on the recommended cutoffs: Low (1–32), Med (33–50), and High (51–99)^21^. Some cases are depicted as HER2-positive given prior HER2-positivity, despite the closest biopsy to T-DXd administration being formally HER2-negative (as suggested by the IHC score). HS-HER2, High Sensitivity-human epidermal growth factor receptor 2; RPPA, Reverse Phase Protein Array; T-DXd, trastuzumab deruxtecan; TTNT, time to next treatment. *did not experience TTNT event.

To do so, we retrieved tissue samples collected prior to T-DXd initiation among patients with MBC treated at the Dana-Farber Cancer Institute, subjecting them to centralized testing with (i) HS-HER2; (ii) RPPA; (iii) HER2DX. Although the same samples were utilized for all cohorts, tissue availability and the specific requirements of each assay ultimately dictated the number of samples available for each analysis. Figure 2 depicts the overlap in data availability and graphically describes the results obtained with the three different tissue-based quantitative HER2 assays. For each sub-cohort, we also separately evaluated the predictive performance of traditional IHC HER2 testing. Finally, next-generation sequencing (NGS) data (Oncopanel) were also retrieved to evaluate whether changes in ERBB2 copy number may be associated with the effectiveness of T-DXd.

**Fig. 3:**
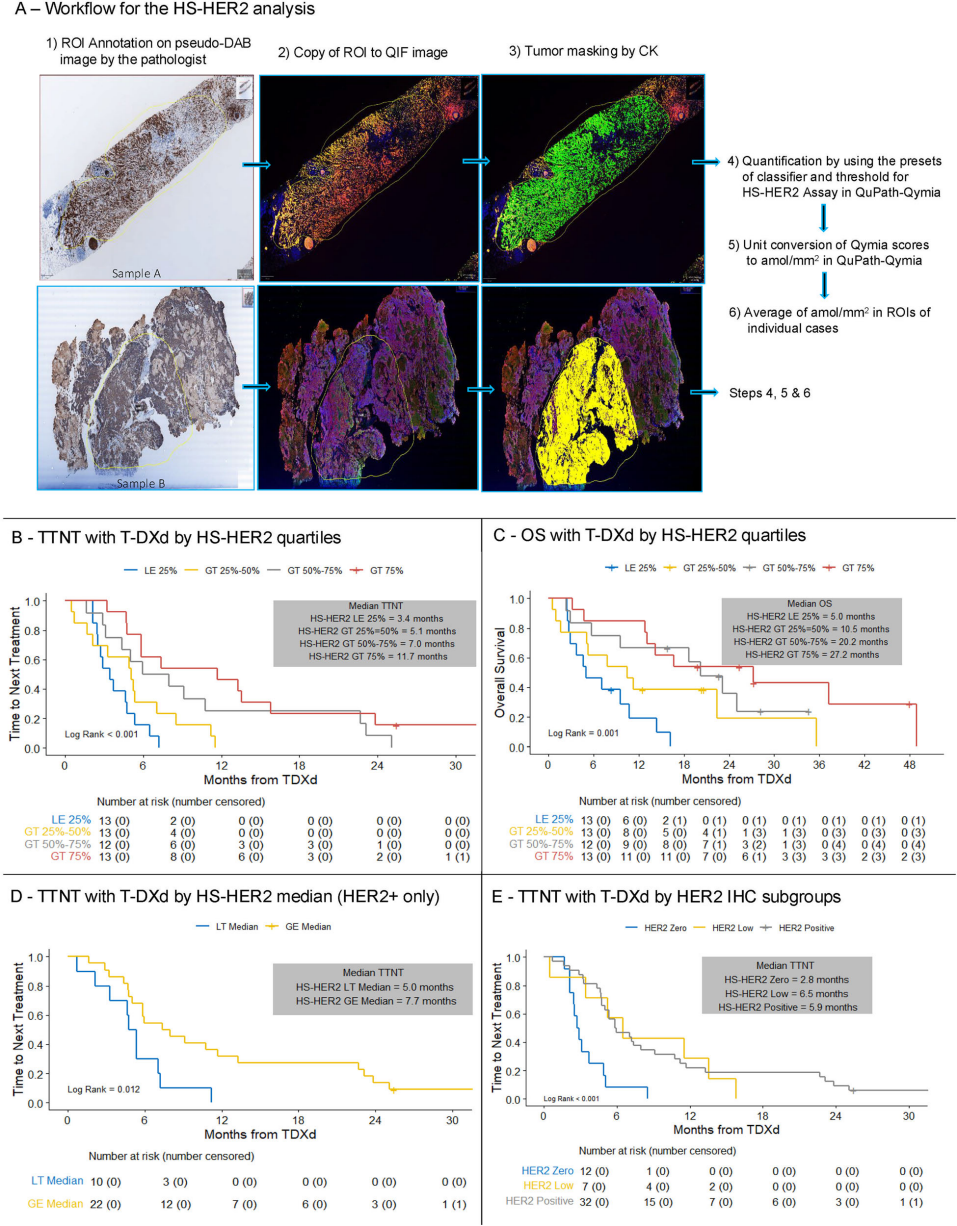
Outcomes with T-DXd according to pre-treatment HS-HER2 status. **A** Workflow for the quantification of HS-HER2; **B** TTNT with T-DXd according to HS-HER2 quartiles; **C** OS with T-DXd according to HS-HER2 quartiles; **D** TTNT with T-DXd according to HS-HER2 median (HER2-positive disease only); **E** TTNT with T-DXd in the HS-HER2 cohort according to the traditional HER2 IHC classification of HER2-positive, HER2-low-HER2-0 in the HS-HER2 population. T-DXd trastuzumab deruxtecan, TTNT time to next treatment, HS-HER2 High Sensitivity-HER2, IHC immunohistochemistry, ROI region of interest, OS overall survival.

We deployed the QuPath-Qymia method of quantitative immunofluorescence on tissue samples collected from 51 patients before the start of T-DXd to determine HS-HER2, able to provide a quantitative measurement of HER2 in attomole/mm^2 (Fig. 3A, Supplementary Table 2). The predictive value of pre-treatment HS-HER2 was assessed as a continuous variable and by quartiles.

Across all 51 patients included in the analysis, HS-HER2 was found to be continuously associated with TTNT (per 5-unit increment hazard ratio [HR] 0.77 [CI: 0.68–0.86], *p* < 0.001) and OS (per 5-unit increment HR 0.79 [95% CI: 0.69–0.90], *p* < 0.001) with T-DXd (Supplementary Tables 3, 4). Additionally, there was a significant association between TTNT and HS-HER2 when divided into quartiles, with a trend of increasing median TTNT (Fig. 3B, *p* < 0.001) and OS (Fig. 3C, *p* = 0.001) with increasing HS-HER2 quartiles. Consistent findings were observed in sub-analyses of patients with HER2-positive MBC (Fig. 3D) and patients with HER2-negative MBC (Supplementary Fig. 3).

Conversely, the standard HER2 IHC subgroups (HER2-positive, HER2-low, HER2-0) sub-optimally predicted outcomes in this subgroup, with comparable outcomes between patients having HER2-low vs. HER2-positive disease (Fig. 3E).

**Fig. 4:**
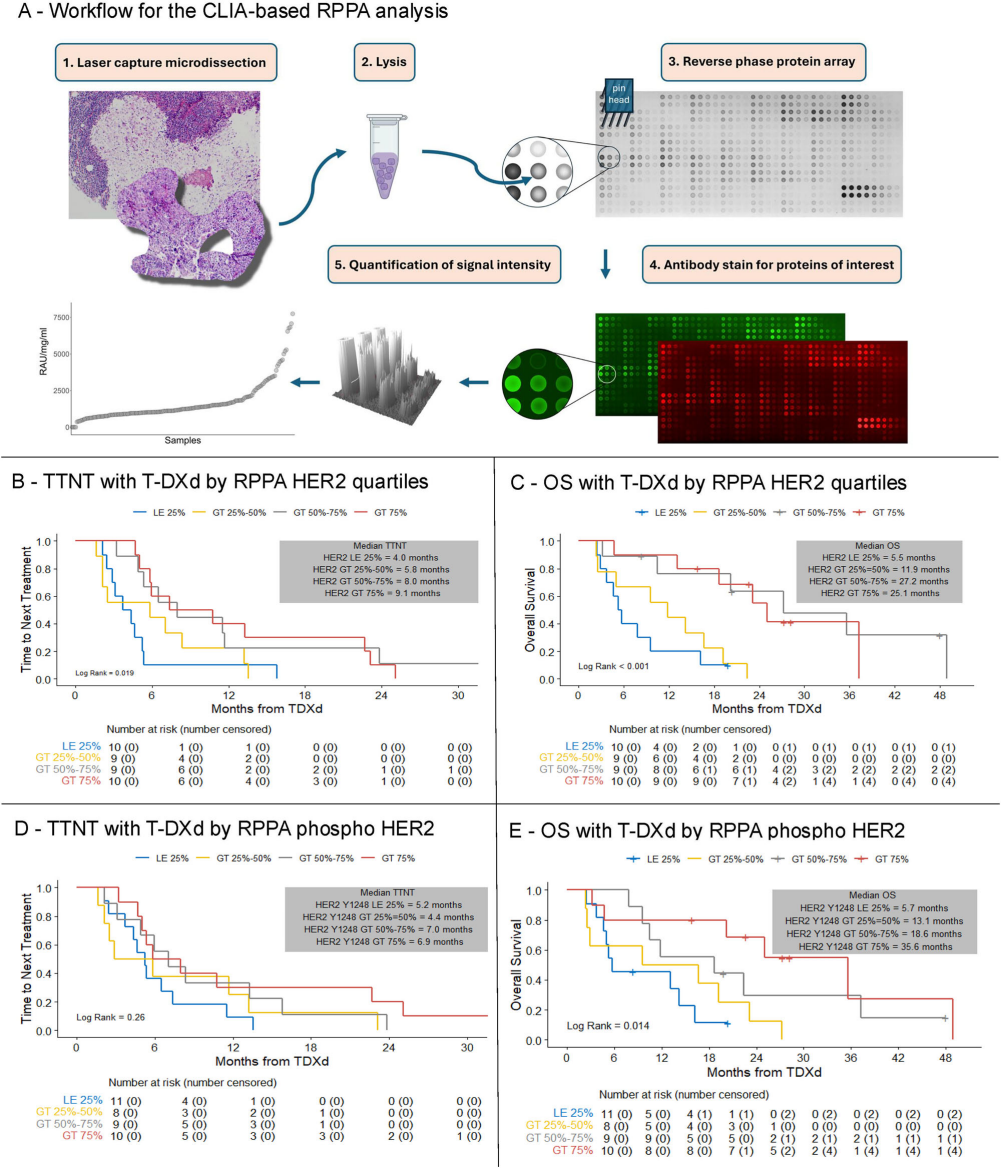
Outcomes with T-DXd according to pre-treatment CLIA-RPPA-based total HER2 protein and HER2 protein activation (phosphorylation) status. **A** Workflow for the CLIA-based RPPA analysis; **B** TTNT with T-DXd according to RPPA quantified total HER2 protein quartiles; **C**. OS with T-DXd according to RPPA HER2 protein expression quartiles; **D** TTNT with T-DXd according to RPPA measured HER2 activation (phosphoHER2 Y1248) quartiles; **E** OS with T-DXd according to RPPA measured HER2 activation (phosphoHER2 Y1248) quartiles; T-DXd trastuzumab deruxtecan, CLIA-RPPA Clinical Laboratory Improvement Amendments- Reverse Phase Protein Array, TTNT time to next treatment, OS overall survival.

To integrate the quantitative evaluation of HER2 with additional markers of potential predictive value, we further analyzed tissue samples from 38 patients (24 with HER2-positive, 14 with HER2-negative disease) via a CLIA-based, multiplex RPPA assay, using lysates derived from laser capture microdissection-enriched tumor epithelium (Fig. 4A, Supplementary Table 5). The RPPA technology uses panels of high-specificity antibodies to enable the simultaneous quantification of several proteins and to assess protein post-translational modifications (e.g., phosphorylation).

Across all patients, the RPPA-based total HER2 expression was found to be directionally associated with TTNT (per 10-unit increment HR 0.95 [95% CI: 0.90–1.01], *p* = 0.08) and significantly associated with OS (per 10-unit increment HR 0.89 [95% CI: 0.82–0.98], *p* = 0.015) (Supplementary Table 6-7). Additionally, there was a significant association between outcomes with T-DXd and HER2 RPPA when divided into quartiles, with a trend of increasing median TTNT and OS with increasing RPPA HER2 quartiles (Fig. 4B, C).

Activation of HER2, as measured by phosphorylation (Y1248) was also found to be directionally associated with TTNT (per 10-unit increment HR 0.89 [95% CI: 0.78–1.02], *p* = 0.087) and significantly associated with OS (per 10-unit increment HR 0.80 [95% CI: 0.66–0.96], *p* = 0.017) (Supplementary Table 8-9), with a trend of increasing median OS with increasing RPPA phosphoHER2 quartiles, whereas the trend was less clear for TTNT (Fig. 4D, E).

Importantly, the mechanism of action of T-DXd involves binding of the ADC to HER2 and subsequent internalization, with intracellular delivery of a TOPO1 inhibitor. Therefore, we hypothesized that the quantification of TOPO1 and the related marker SLFN11 may also enable to predict the activity of T-DXd.

**Fig. 5:**
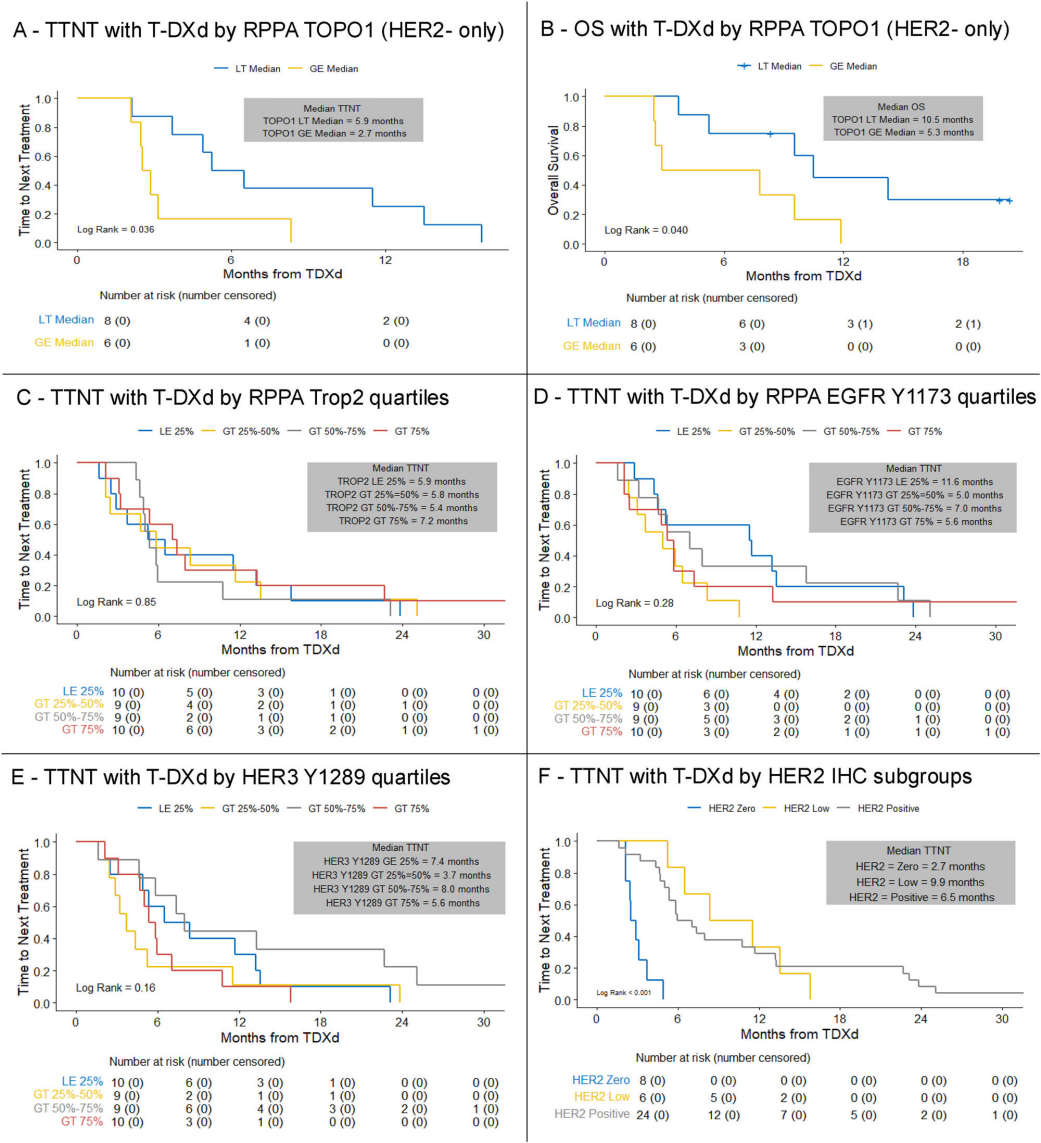
Outcomes with T-DXd according to pre-treatment CLIA-RPPA-based quantitative status of markers beyond HER2. **A** TTNT with T-DXd according to RPPA measured TOPO1 protein expression median (HER2-negative only); **B** OS with T-DXd according to RPPA measured TOPO1 protein expression median (HER2-negative only); **C** TTNT with T-DXd according to RPPA Trop2 quartiles; **D** TTNT with T-DXd according to RPPA measured EGFR protein expression quartiles; **E** TTNT with T-DXd according to RPPA measured phosphoHER3 protein expression quartiles; **F** TTNT with T-DXd according to the traditional HER2 IHC classification of HER2-positive, HER2-low-HER2-0 in the RPPA population. T-DXd trastuzumab deruxtecan, TTNT time to next treatment, RPPA Reverse Phase Protein Array, OS overall survival.

Neither TOPO1 nor SLFN11 expression was found to be significantly associated with TTNT in the overall cohort (Supplementary Table 10, 11), possibly due to the lack of prediction in the HER2-positive cohort, where the activity of T-DXd may be predominantly driven by the overexpression of HER2. However, when the analysis was restricted to patients with HER2-negative disease, TOPO1 expression was found associated with outcomes, with higher expression of TOPO1 found significantly associated with worse TTNT and OS with T-DXd (Fig. 5A, B, Supplementary Table 11). A similar effect was noted for SLFN11, with a significant continuous association with outcomes in the HER2-negative cohort (Supplementary Table 10).

In an exploratory fashion, we also analyzed the correlation between other ADC targets and the effectiveness of T-DXd. In line with the absence of binding of T-DXd to such targets, no clear association was observed between outcomes with T-DXd and the RPPA-based measurement of Trop2, phosphorylated EGFR or phosphorylated HER3 (Fig. 5C-E).

We finally evaluated the predictive performance of conventional HER2 IHC in the RPPA sub-cohort. Among the 38 patients included in the RPPA-based proteomic analysis, the standard HER2 IHC subtypes (HER2-positive, HER2-low, HER2-0) sub-optimally predicted outcomes, with patients having HER2-low disease unexpectedly demonstrating longer TTNT than those with HER2-positive disease (Fig. 5F).

**Fig. 6:**
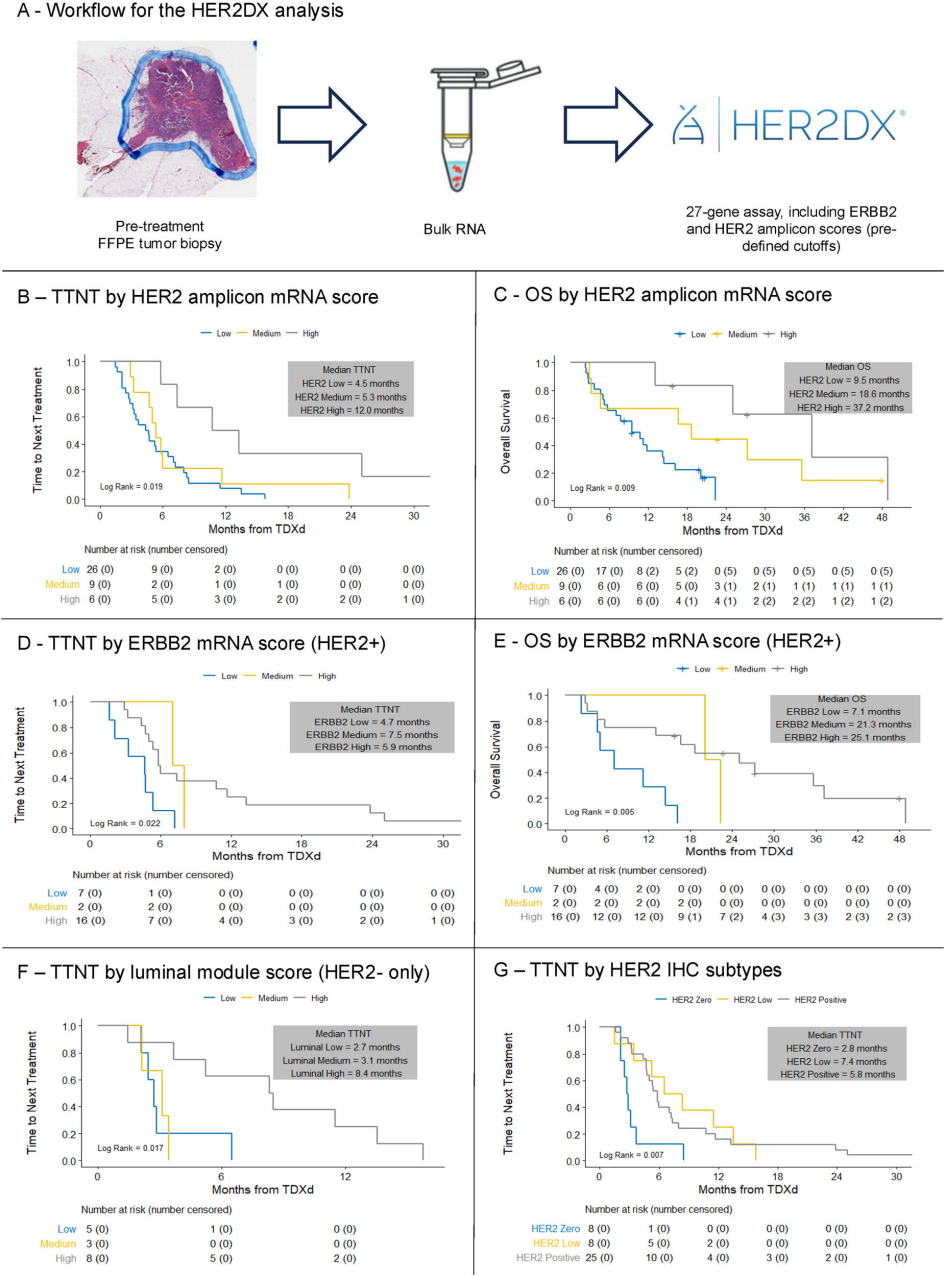
Outcomes with T-DXd according to pre-treatment HER2DX modules. **A** Description of HER2DX gene expression modules; association between the HER2 amplicon module with TTNT (**B**) and OS in all patients (**C**); association between the HER2 amplicon module with TTNT (**D**) and OS in HER2-positive MBC (**E**); **F** association between TTNT and the luminal module in patients with HER2-negative disease; **G** TTNT with T-DXd according to the traditional HER2 IHC classification of HER2-positive, HER2-low-HER2-0 in the HER2DX population. TTNT time to next treatment, OS overall survival, MBC metastatic breast cancer, T-DXd trastuzumab deruxtecan, IHC immunohistochemistry.

Pre-T-DXd tissue samples from 41 patients (25 with HER2-positive, 16 with HER2-negative disease, Supplementary Table 12) were analyzed with the standardized HER2DX gene expression assay, with analysis of different transcriptomic modules (Fig. 6A).

The HER2 amplicon signature (combining the expression of *ERBB2*, *GRB7*, *STARD3* and *TCAP*) was found to be significantly associated with TTNT (per 1-unit increment HR 0.70 [95% CI: 0.56–0.87], *p* = 0.001) and OS (per 1-unit increment HR 0.65 [95% CI: 0.5–0.84], *p* = 0.001) with T-DXd, including in subgroup analyses of HER2-positive and HER2-negative MBC (Supplementary Table 13, 14). Additionally, there was a significant association between TTNT and the HER2DX HER2 amplicon module when divided according to pre-established thresholds^21^, with a significant increase in median TTNT (*p* = 0.019, Fig. 6B) and OS (*p* = 0.009, Fig. 6C) with increasing HER2 amplicon scores, including when restricting to patients with HER2-positive disease (Supplementary Table 13-14).

A significant continuous association was also observed between TTNT and the expression of *ERBB2* (*p* = 0.002) (Fig. 6D, E, Supplementary Tables 15, 16), whereas no significant association was observed with the IGG module (*p* = 0.29), the proliferation module (*p* = 0.86) and the luminal module (*p* = 0.32) when evaluated continuously, although a trend towards better TTNT was observed with higher expression of luminal genes among patients with HER2-negative disease (Fig. 6F).

Among the 41 patients included in the HER2DX analysis, the standard HER2 IHC subtypes (HER2-positive, HER2-low, HER2-0) sub-optimally predicted outcomes, with comparable outcomes between patients having HER2-low vs. HER2-positive disease (Fig. 6G).

NGS data were retrieved for 53 patients with HER2-negative MBC at T-DXd start, including 36 (67.9%) with hormone receptor-positive/HER2-negative and 17 (32.1%) with triple-negative disease. A total of 8 patients (15.1%) exhibited *ERBB2* heterozygous loss (6 with hormone receptor-positive disease, 2 with triple-negative disease). A trend towards shorter TTNT (4.9 months vs. 7.6 months, *p* = 0.60, Supplementary Fig. 4A) and OS (8.0 vs. 11.4 months, *p* = 0.55, Supplementary Fig. 4B) was observed among patients harboring *ERBB2* heterozygous loss.

In summary, quantitative HER2 analyses on tissue samples collected prior to starting treatment with T-DXd enabled to significantly predict the real-world activity of T-DXd. Higher HS-HER2, RPPA HER2 (or phosphorylated HER2), and ERBB2 (or HER2 amplicon) mRNA expression were all found associated with outcomes with T-DXd, whereas, in each biomarker sub-cohort, traditional testing of HER2 with IHC sub-optimally predicted the activity of T-DXd. In addition, among patients with HER2-negative MBC, high RPPA expression of TOPO1 and the presence of ERBB2 heterozygous loss on NGS were found associated with worse outcomes with T-DXd, although both findings warrant caution in interpretation, since based on small subgroups of patients and given lack of statistical significance for the NGS analysis.Of note, although tissue-based HER2 quantification has proven promising in predicting outcomes with T-DXd, it requires availability of tumor tissue, the collection of which often involves invasive techniques. The evaluation of ctDNA enables to overcome such invasiveness, enabling the characterization of tumor biology from blood draws. Moreover, it can more comprehensively capture the temporal heterogeneity of tumors compared to tissue-based analyses.

**Fig. 7:**
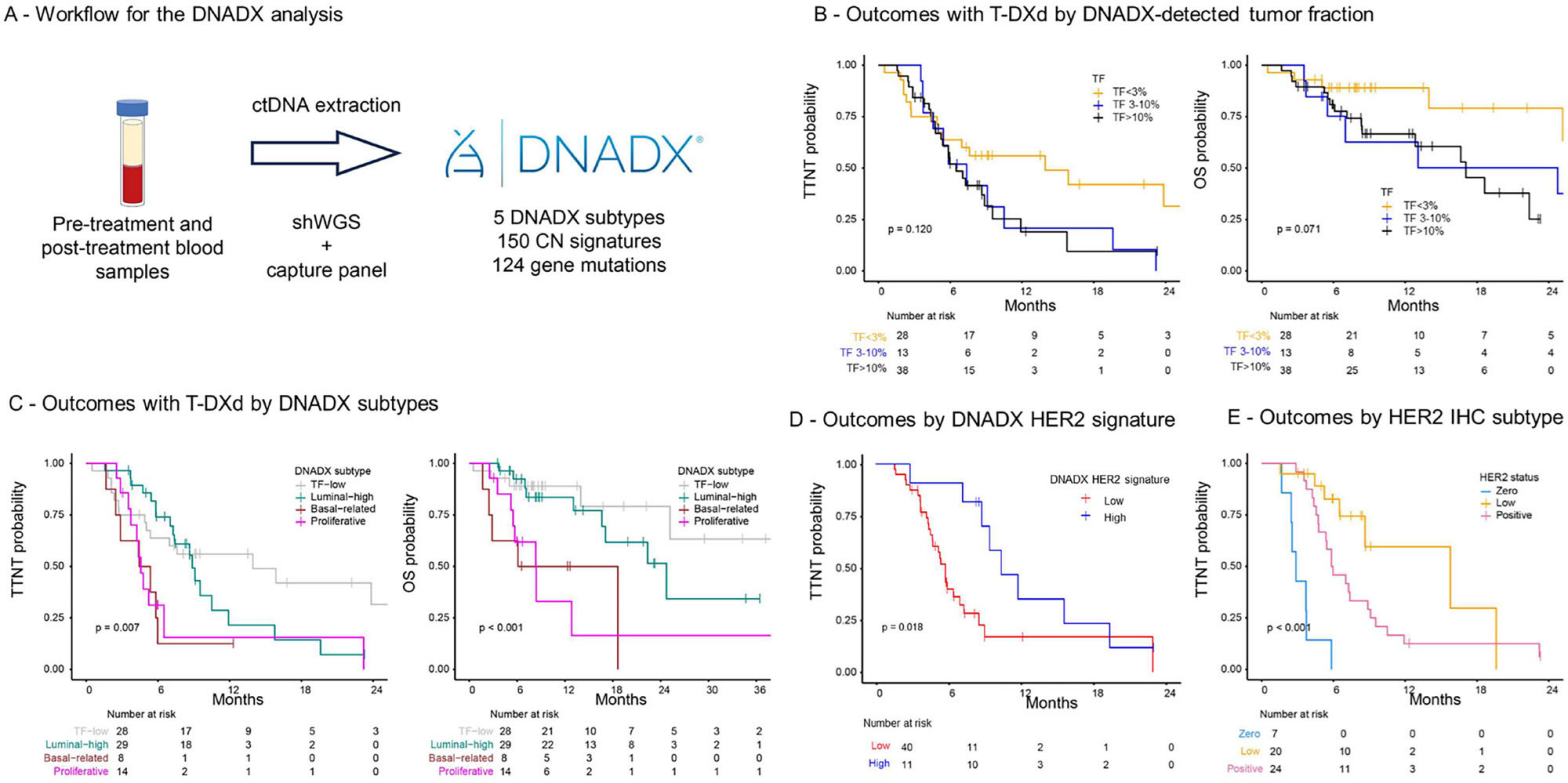
Prediction of outcomes with T-DXd using DNADX. **A** Description of DNADX workflow; **B** Outcomes with T-DXd for metastatic breast cancer according to DNADX-detected tumor fraction; **C** Outcomes with T-DXd for metastatic breast cancer according to DNADX subtype; **D** Outcomes with T-DXd for metastatic breast cancer according to the DNADX HER2 signature; **E** Outcomes with T-DXd for metastatic breast cancer according to HER2 IHC status in the cohort of patients with detectable tumor fraction. T-DXd trastuzumab deruxtecan, TTNT, time to next treatment, OS overall survival.

To evaluate the predictive potential of a plasma-based assessment of tumor biology, we conducted ctDNA testing with the novel DNADX assay on a total of 140 plasma samples from 98 patients treated with T-DXd (Fig. 7A). Among the available pre-T-DXd plasma samples, the median tumor fraction (TF) was 9% (range: 0–63%), with 28 patients (35%) having a TF of 0%, and 51 (65%) having a detectable TF ≥ 3%. DNADX classified the baseline samples into 5 cluster subtypes: Cluster-0 (TF = 0%, *n* = 28, 35%); Cluster-1 (copy number-low, 0%); Cluster-2 (luminal-like, *n* = 29, 37%); Cluster-3 (basal-like, *n* = 8, 10%); and Cluster-4, (proliferative, *n* = 14, 18%).

We observed associations between ctDNA tumor fraction and DNADX cluster subtypes with the activity of T-DXd. Indeed, patients having undetectable TF trended towards better outcomes compared to patients with detectable TF (Fig. 7B). By adopting the TF-low group as reference, both the Basal and Proliferative groups experienced worse TTNT (hazard ratio 3.55 and 3.05 respectively, *p* < 0.05 for both) and worse OS (hazard ratio 1.88 and 1.68, respectively, *p* < 0.05 for both), whereas outcomes were not different for the Luminal-high group.

Notably, the DNADX cluster subtype distribution was found significantly associated with both TTNT and OS (Fig. 7C; *p* = 0.007 and *p* < 0.001, respectively).

We then focused on a blood-based prediction of HER2-dependency of the tumors. The plasma-based, DNADX HER2 signature was calculated on baseline samples from 51 patients with detectable (>3%) TF (8.6% HER2-0, 24.7% HER2-low, 29.6% HER2-positive). We observed a significant association of the HER2 signature with TTNT (*p* = 0.045), particularly when comparing patients with DNADX HER2-signature-low vs. HER2-signature-high disease (Fig. 7D; HR = 2.64; p = 0.0152), whereas IHC was less predictive of benefit in this population, with longer TTNT in HER2-low tumors compared with HER2-positive or HER2-0 tumors (Fig. 7E).

The most frequent mutations observed in pre-treatment samples were in *TP53* (26%), *MYC* (17%), *KMT2C* (17%), *PIK3CA* (17%), *GATA3* (14%), *ATM* (12%), *ERBB2* (11%), and *ESR1* (11%) (Supplementary Fig. 5A). Baseline mutations in *TP53* were significantly associated with worse TTNT with T-DXd in univariable (HR = 3.4, *p* < 0.001) and multivariable analysis after adjusting by DNADX subtype and DNADX HER2 signature (HR = 4.02; *p* < 0.001).

The most frequent mutations observed in post-treatment samples were in *TP53* (26%), *PIK3CA* (26%), *TERT* (20%), *ARID1B* (20%), *ATM* (19%), *CDH1* (17%), and *ESR1* (15%) (Supplementary Fig. 5B). *ARID1B* mutations were significantly enriched in post-treatment samples (odds ratio=5.88; *p* = 0.009). Mutations in *NFE2L2, HCFC2, GNA11, FGFR1*, and *USP9X* were only detected in post-treatment samples (Supplementary Fig. 5C).

In summary, a blood-based evaluation of tumor biology with DNADX enabled to identify populations of patients with MBC with distinct outcomes, and the profiling of pre- and post-T-DXd ctDNA unveiled a negative prognostic effect of pre-treatment *TP53* mutations and an enrichment in *ARID1B* mutations after treatment with T-DXd.

## Discussion

In the present work, we addressed three key unmet needs which currently challenge the utilization of T-DXd for MBC: the paucity of real-world data in US patients, the absence of data on the predictive value of HER2-low dynamics, and the lack of effective biomarkers able to guide treatment with T-DXd.

Herein, we show that T-DXd exhibits relevant real-world activity for the treatment of MBC, although with relevant divergence from clinical trial data. Indeed, compared with the PFS described in the DESTINY trials (17–29 months in HER2-positive^4^, 10–13.2 months in HER2-low MBC^2,3^), we observed a TTNT of 10.4 months for HER2-positive and 7.6 months for HER2-low MBC. This is highly consistent with what was reported in the DAISY phase 2 trial^13^ and in other real-world analyses^22^, and possibly related to differences in prior lines of treatment between our cohort and clinical trial populations.

Furthermore, in the present study we report significant differences in the performance of T-DXd depending on the dynamics of HER2-low expression: the longest activity (TTNT 9.4 months) was observed among patients who maintained HER2-low disease both in the primary and metastatic settings, whereas shorter TTNT (3.0–5.6 months) was observed among patients with changes in HER2 status (HER2-low to HER2-0 or vice versa), highlighting the clinical relevance of HER2 temporal and spatial heterogeneity.

Additionally, to address the limitations of IHC, which has proven insufficient in predicting the efficacy of ADCs^2,3,12,23^, we explored multiple novel biomarkers through comprehensive analyses of HER2 expression on tissue and plasma samples collected before T-DXd, showing that the quantitative assessment of HER2 can continuously predict the efficacy of T-DXd. The quantitative immunofluorescence-based HS-HER2 was found continuously associated with outcomes with T-DXd, including in subgroup analyses of HER2-positive and HER2-negative MBC. Similarly, RPPA-based quantification of both total levels of HER2 and activation of HER2 (phospho-HER2 at Y1248) was found significantly associated with OS after T-DXd, although the association with TTNT was not statistically significant. Moreover, the quantification of the HER2 (and HER2 amplicon) mRNA via HER2DX was also found continuously associated with the activity of T-DXd. The multiplex capabilities of the RPPA-based proteomic panel enabled concomitant quantification of biomarkers associated with payload sensitivity: most notably, higher TOPO1 expression was found to be associated with shorter TTNT and OS in HER2-negative MBC, suggesting that additional aspects of ADC activity beyond the antibody target may further refine prediction of activity of T-DXd. Lastly, pre-treatment ctDNA profiling with DNADX enabled evaluation of a HER2 signature that showed a strong association with T-DXd efficacy. Confirmation of this latter finding may provide a non-invasive method for the prediction of T-DXd efficacy in MBC. Overall, each of the assays tested in this pilot study (HS-HER2, multiplex RPPA, HER2DX, DNADX) demonstrated some degree of association with outcomes with T-DXd. Although the small numbers prevent deriving strong conclusions on which assay is most promising, a validation of these results in a larger cohort is ongoing and is expected to provide helpful data to more clearly focus future validation efforts. Besides the data on outcome prediction, logistic and financial aspects are also expected to play a role in determining which assays will be feasible to implement in the clinic.

We further analyzed the genomic landscape of MBC before and after treatment with T-DXd. We show that *ERBB2* heterozygous loss is a relatively common event in HER2-negative MBC (15.1% of the patients in our study), and it is associated with numerically worse outcomes compared with patients having wild-type *ERBB2*. This is in line with a prior report from DAISY, where most patients harboring *ERBB2* heterozygous loss (*n* = 4/6) did not respond to T-DXd^13^; as well as with a more recent real-world analysis from the ClinicoGenomic Database, where 11/95 (11.6%) patients with HER2-low MBC had ERBB2 heterozygous loss and were found to experience significantly worse outcomes with T-DXd.^24^ We also observed enrichment in ARID1B mutations and emergence of mutations in NFE2L2, HCFC2, GNA11, FGFR1, and USP9X in post-T-DXd ctDNA samples, with their functional relevance to be further investigated in future studies.

Key limitations of our study include its retrospective nature, the limited sample size of the translational analyses with no conduction of false discovery rate adjustment and the absence of prospectively collected metrics of T-DXd efficacy, such as PFS by RECIST 1.1. However, the numerical concordance of the outcomes observed in our study with prior studies^13,22^ suggests the consistency of our results. Moreover, a significant strength of our study is the consistent and meaningful association of quantitative HER2 testing via multiple different orthogonal assays with T-DXd outcomes. Our results appear to be biologically rational as the association of the quantitative HER2 values with T-DXd outcomes appeared to the specifically oriented to HER2, whereas the quantification of other HER family members (phospho-EGFR, phospho-HER3) showed less or no association with outcomes. Moreover, we saw no association between outcomes with T-DXd and other ADC markers (e.g., Trop2), or unrelated markers (IGG and proliferation HER2DX modules).

In conclusion, T-DXd demonstrated encouraging real-world outcomes for the treatment of MBC, particularly for patients with HER2-positive or stable HER2-low disease. The quantitative pre-T-DXd assessments of HER2 with highly sensitive proteomic (HS-HER2, CLIA-RPPA), transcriptomic (HER2DX) or liquid biopsy (DNADX) assays were found significantly associated with the performance of T-DXd. Validation studies are ongoing to clarify the improvement in outcome prediction with these novel assays and to further assess the added value of multiplexing for additional ADC-relevant markers.

## Methods

We retrospectively analyzed data for patients with MBC who received T-DXd at Dana-Farber Cancer Institute (DFCI) (Boston, MA) between July 1, 2017, and January 20, 2023 (*n* = 161 patients) and Duke Cancer Institute (Durham, NC) between March 31, 2020 and April 30, 2022 (*n* = 30 patients). HER2, estrogen receptor (ER), and progesterone receptor (PR) expression levels were abstracted from pathology records. Patients were categorized as having HER2-positive disease if they had any biopsy testing HER2 IHC 3+ or In Situ Hybridization (ISH) amplified at any timepoint prior to T-DXd; this subgroup included patients with potential temporal changes in HER2 status, which may have led to treatment following both the HER2-positive and HER2-negative algorithms. Patients classified as HER2-negative were further divided into HER2-low or HER2-0 based on the most recent biopsy prior to the start of T-DXd.

We determined TTNT, OS, toxicities, TTNT based on changes in HER2 status, and TTNT with post-T-DXd regimens. OS was defined as time from the initiation of T-DXd treatment to death or censored at the last known vital status date. TTNT was defined as the time interval between the initiation of treatment with T-DXd and the initiation of a subsequent regimen or death or censored at the last known vital status date. Detailed review of the medical records was performed to determine the incidence, grade, management and outcomes of key toxicities with T-DXd. Pre-treatment samples were profiled via proteomic (HS-HER2^14^), a commercially available CLIA Reverse Phase Protein Array-based proteomic breast cancer assay [RPPA^15^]), genomic (OncoPanel^18,20^), transcriptomic (HER2DX^16^) and plasma-based (DNADX^17^) assays. Pre-treatment tissue samples were sectioned according to a pre-specified priority list, with slides submitted for testing with (1) HS-HER2, (2) RPPA, (3) HER2DX. The limited tissue availability for certain samples accounted for slight differences in the final number of samples available for each analysis. For subset analyses for the three different tissue-based biomarkers (HS-HER2, RPPA, and HER2DX), TTNT and OS were associated with each of the biomarker expression (in continuous scale, split by median, and grouped into tertiles or quartiles. Medians and quartiles were applied whenever no prior cutoff method had been established in prior studies). Associations between categorical variables and TTNT or OS were evaluated using Kaplan-Meier plots and a global log-rank test implemented in the survival package in R. For continuous variables, a Cox proportional hazards model was fit and the Wald test *p*-value for the coefficients is reported. Follow-up was estimated using reverse Kaplan-Meier.

To explore resistance mechanisms to T-DXd, we also compared genomic alterations on ctDNA before and after treatment with T-DXd by using the DNADX assay. To determine quantitative HER2 protein, we developed an analytic assay based on protein concentrations in cell line standards, determined by mass spectrometry. This method is described previously.^14^ In brief, cell line microarrays (CMA) composed of 5 cell lines (including JURKAT #HTB-152, BT20 #HTB-19, T47D #HTB-133, ZR-75-1 #CRL-1500 and BT483 #HTB-121 created by Array Science LLC) and patient tissue slides were offline baked at 60 *°*C for at least 1 h prior to autostaining. One CMA is processed with a maximum of 9 patient tissue slides in each BOND slide tray. The Leica BOND Rx autostainer protocol is as follows: deparaffinization with BOND dewax solution (AR9222), antigen retrieval with BOND HIER epitope retrieval solution 2 (AR9640) at 97 *°*C for 20 min, blocking with ReadyProbes Endogenous HRP & AP blocking solution (R37629, Invitrogen) for 10 min and with BSA for 30 min, 1 h incubation with primary rabbit monoclonal HER2 antibody (clone 29D8, #2165, IgG, Cell Signaling) at optimal concentration of 1ug/ml mixed together with 1:100 concentration of pan-CK (Clones AE1/AE3, REF#M3515, Dako), amplification with Rabbit Envision+ System–HRP labeled polymer anti-Rabbit (K400311-2, Dako) mixed together with 1:100 dilution of a green-fluorescent Alexa Fluor 546 Goat-anti-Mouse IgG (H + L) Cross-Adsorbed Secondary Antibody (REF#A11003) for 1 h, staining with 1:50 dilution of a red-fluorescent Tyramide Signal Amplification (TSA) Cyanin 5 (SAT705A001EA, Akoya Biosciences) for 10 min and nuclear staining with 1:500 dilution of a blue-fluorescent 4′,6-diamidino-2-phenylindole (DAPI) for 10 min. Subsequently, the excess DI water on each slide was wiped off carefully and coverslipped with ProLong Gold Antifade mounting reagent (P36930, Invitrogen).

The HS-HER2 assay was successful on 71 pre-T-DXd treatment tissue samples which were 5 um sections from formalin-fixed, paraffin-embedded (FFPE) tumors (either resections or biopsies). In total, HS-HER2 data was obtained for 53 patients, including the primary tumor from 20 patients (39.2%), a metastatic biopsy in 15 (25.5%), both in 18 patients (35.3%). After exclusion of 2 patients for lack of adequate follow-up, the analysis included a total of 51 patients (32 with HER2-positive, 19 with HER2-negative disease)

The control CMAs and pre-T-DXd samples were scanned at 20× magnification on the Rarecyte CyteFinder HT II multiplexed fluorescent imaging platform (RareCyte, serial number: HT-0452201). After the patient tissue slide was scanned, a pseudo-IHC image was generated and representative regions of interest (ROIs) of each biopsy were selected by a board-certified pathologist. Three cases were omitted from further analysis due to their insufficient or damaged tumor tissue. Then, using the CMA standard (calibrated by liquid chromatography-tandem mass spectrometry (LC-MS/MS) of each cell line), the signal intensity in the ROI was converted to amol/mm^2^.

We evaluated the association of HS-HER2 in the closest samples collected prior to T-DXd with TTNT and OS in continuous terms and by quartiles of HS-HER2. Cox proportional hazards models were utilized to estimate hazard ratios, and log-rank test *p*-values were reported. On top of that, Kaplan-Meier method was used to calculate median estimates. Additionally, Weighted Cohen’s kappa statistic was calculated to assess the agreement in HS-HER2 score between primary and metastatic samples for patients that had both samples.

In total, 47 FFPE tumor tissue specimens were available for RPPA-based proteomic analysis, three of which did not contain a sufficient quantity of tumor cells and were removed from further analysis. In total, 44 samples from 38 patients were successfully analyzed. Specimens were serially sectioned at 8 µm and stored at 4 °C for up to 292 days prior to microdissection.

Sections were stained with hematoxylin, followed by tumor epithelial cell enrichment (~5–10 um^2^) via laser capture microdissection using an ArcturusXT laser capture microdissection system (Molecular Devices, LLC, San Jose, CA, USA). Samples were stored at -80°C prior to lysis. Samples were lysed by heating a solution of 225 mM tris hydrochloride (Rockland Immunochemicals, Pottstown, PA, USA), 50 mM tris(2-carboxyethyl)phosphine (TCEP) (ThermoFisher, Waltham, MA, USA), 10% glycerol (FisherScientific, Hampton, NH, USA), and 4% SDS (FisherScientific) to 95 °C for 20 min, followed by a 2 hour water bath incubation at 80°C, and then centrifuged for 15 min.

RPPA-based protein/phosphoprotein analysis was performed as previously described^25,26^ in a CLIA-certified and College of American Pathologists (CAP)- accredited laboratory. In short, lysates were diluted to a concentration of 250 ug/mL and stored at -80°C before printing. Samples were printed in triplicate at 9nL per spot onto nitrocellulose backed slides (Grace Biolabs, Bend, OR, USA) using a Quanterix 2470 arrayer (Quanterix, Billerica, MA, USA). Positive and negative control cell lysates, bovine serum album (BSA) standards, and analyte-specific calibrator curves were printed alongside samples for quality control purposes.

Prior to staining, nitrocellulose slides were pre-treated with ReBlot (MillliporeSigma, Rockville, MD, USA) and blocked with I-Block (Applied Biosystems, Waltham, MA, USA). Nitrocellulose slides were probed with the respective primary antibodies (EGFR T654, Abcam ab75986; EGFR Y1068, Cell Signaling Technology (CST) 3777; EGFR Y1173, CST 4407; HER2, Thermo Fisher Scientific MA5-14509; HER2 Y1248, Abcam ab201013; HER3 Y1289, CST 4791; SLFN11, CST 34858; TOPO1, Abcam ab109374; TROP2, CST 47866) for 30 minutes, followed by secondary antibody incubation using biotinylated goat anti-rabbit IgG (H + L) (BA1000, Vector Laboratories, Newark, CA, USA). Signal amplification and staining was performed using tyramide based avidin/biotin amplification, followed by a streptavidin-conjugated IRDye 800 CW (LI-COR, Lincoln, NE, USA). Negative control slides were stained in the absence of the primary antibody. Total protein deposition was measured using Fast Green FCF (FisherScientific). Slides were scanned using an InnoScan 710IR scanner (Innopsys, Carbonne, France) and spot intensities were measured using Mapix software (Innopsys).

Total protein content per sample was calculated using a BSA standard curve printed on the same slide. Background and negative subtracted intensity values were fit to an analyte-specific calibrator and total protein normalized. Antibodies used for immunostaining were validated for major band with appropriate molecular weight using western blotting as previously described.^25,27^ We evaluated the association of different biomarkers (total HER2, phosphoHER2, TOPO1, SLFN11, Trop2, phosphoHER3, and phosphoEGFR) with TTNT and OS in continuous terms and categorized by quartiles of each biomarkers. Cox proportional hazards models were utilized to estimate hazard ratios, and log-rank test p-values were reported. On top of that, Kaplan-Meier method was used to calculate median estimates.

RNA samples were extracted from 41 FFPE tumor tissue samples using the ReliaPrep FFPE Total RNA Miniprep System (Promega) following manufacturer’s protocol. From FFPE RNA, the HER2DX standardized assay was centrally performed in Reveal Genomics (Barcelona, Spain) as previously described.^28,29^

The HER2DX assay is based on 4 different gene comprising 27 genes, including the 14-gene immunoglobulin (IGG) module (i.e., *CD27, CD79A, HLA-C, IGJ, IGKC, IGL, IGLV3-25, IL2RG, CXCL8, LAX1, NTN3, PIM2, POU2AF1, TNFRSF17*), a 4-gene tumor cell proliferation signature (*EXO1, ASPM, NEK2, KIF23*), a 5-gene luminal differentiation signature (*BCL2, DNAJC12, AGR3, AFF3, ESR1*), and the 4-gene HER2 amplicon signature (*ERBB2, GRB7, STARD3, TCAP*). For each signature, the normalized gene expression was calculated for each patient. The HER2DX ERBB2 score was calculated based on the *ERBB2* mRNA levels. Pre-established cutoffs were used for each signature/variable. Missing data was not imputed. We evaluated the association of different biomarkers (HER2 amplicon, ERBB2 gene, IGG, luminal, and proliferation) with TTNT and OS in continuous terms and categorized by pre-established thresholds of each biomarker. Cox proportional hazards models were utilized to estimate hazard ratios, and log-rank test p-values were reported. On top of that, the Kaplan-Meier method was used to calculate median estimates.

OncoPanel is a targeted NGS assay that detects genomic alterations in tissue samples, including insertions, deletions, single-nucleotide variants, copy number alterations, and structural variants across a panel of 447 genes with evidence as drivers of cancer biology.^18,20^ OncoPanel data were retrieved for patients that received clinical NGS testing during the course of their disease. Analyses were restricted to patients with HER2-negative disease. We compared TTNT and OS among patients with presence vs. absence of *ERBB2* heterozygous loss.

A total of 81 pre-treatment and 59 post-treatment plasma samples collected from patients with MBC treated with T-DXd were subject to DNADX testing. Of the pre-treatment samples, 66 (81.5%) were collected within 6 months before starting T-DXd and 15 (18.5%) were collected more than 6 months before T-DXd initiation.

cfDNA was obtained from 3 mL of plasma using the QIAamp Circulating Nucleic Acid Kit (QIAGEN Inc.) according to the manufacturer’s instructions and quantified with a Qubit dsDNA high-sensitivity assay kit and the Qubit 4.0 fluorometer (Life Technologies, Carlsbad, CA, USA). cfDNA was concentrated using SpeedVac to fulfill the requirements for library preparation. Library preparation was performed by ligating unique dual indexes (UDI) custom adapters coupled to Unique Molecular Identifiers (UMI) to a minimum of 10 ng of the isolated cfDNA (10-50 ng dsDNA). More specifically, the fragment ends of cfDNA were blunted and 5’ phosphorylated and, after that, 3’ ends were A-tailed to favor adapter ligation. Adapters were 10 bp—UDI as recommended to mitigate errors introduced by index-hopping or switching in Illumina instruments with patterned flow cells, such as the NovaSeq 6000. Indexed libraries were quantified by qPCR using the KAPA Library Quantification Kit (Roche Sequencing Solutions), pooled, and sequenced in a NovaSeq 6000 Illumina at 1x mean coverage with read length of 2 ×150 bp. ShWGS was analyzed with hmmcopy_utils (https://github.com/shahcompbio/hmmcopy_utils) and ichorCNA v0.2.0 (https://github.com/broadinstitute/ichorCNA), with a bin size of 500 kb and default parameters.^30^

A total of 150 DNA-based phenotypic signatures, including the DNADX HER2 signature, and 5 DNADX subtypes (Clusters-0 [TF = 0], −1 [copy number-low], −2 [luminal-like], −3 [basal-like] and −4 [proliferative]), were identified as previously described.^17,31^ Briefly, DNA-sequencing segmentation files from ichorCNA output were first mapped to gene-level features. The signal of 519 DNA segments was calculated using the mean copy number score across genes within each segment. The coefficients of DNA segments for predicting gene signatures were obtained from Xia et al. DNA-based signature scores were calculated as the weighted average of DNA segment values for each sample: a final signature score was obtained by adding all values (i.e., coefficient of segment A x signal of segment A plus coefficient of segment B x signal of segment B)^31^. The 5 DNA-based subtypes or clusters were identified using a previously reported DNA-based subtype predictor, which is based on unsupervised analysis of tumor samples and the 150 DNA-based signatures.^17^ For all samples with the 150 DNA-based signatures available, we calculated the Euclidean distances to the 4 centroids and assigned a cluster class to each sample based on the nearest centroid.

For TF analyses, the selected cutoffs are based on the validated performance characteristics of ichorCNA: a TF < 3% corresponds to the established limit of detection, whereas the 10% cutoff is used as an operational threshold that robustly represents high TF values.

ctDNA was processed for library preparation using a custom hybridization-based capture panel targeting 124 genes with reported somatic mutations, performed with Agilent SureSelectXT Low Input Target Enrichment System (Agilent Technologies, Inc). Indexed libraries were quantified by qPCR using the KAPA Library Quantification Kit (Roche Sequencing Solutions), pooled and sequenced in a NovaSeq 6000 Illumina (2 x 100 bp) at an average coverage of 500×. Frequent single-nucleotide polymorphisms in the population were removed based on the gnomAD database (allele frequency ≤0.0001). Data were manually curated, and classification of identified variants was performed using publicly available databases (COSMIC, cBioPortal, ClinVar, VarSome, OncoKB).

### Ethics

All research was performed in accordance with the Declaration of Helsinki. Institutional review board approval for the conduct of this study was obtained from the Dana-Farber/Harvard Cancer Center and from the Duke Cancer Institute. Written informed consent was obtained from all participants.

## Supplementary information

Supplementary

## Data Availability

Data can be requested from the corresponding authors for academic use, subject to approval of a research plan, a data transfer agreement and ethics committee approval.
