# Supplementary material for: Quantitative HER2 tissue and plasma profiling predicts the activity of trastuzumab deruxtecan for breast cancer
Source: NPJ Precis Oncol. 2026 Mar 13;10:141. doi: 10.1038/s41698-026-01365-6 (PMC13046964; doi:10.1038/s41698-026-01365-6)
Supplement: Supplementary file 1 — Supplementary [file 41698_2026_1365_MOESM1_ESM.pdf]

Supplement: Quantitative HER2 tissue and plasma profiling predicts the activity of trastuzumab deruxtecan for breast cancer

- **Supplementary Figure 1.** TTNT with regimens administered immediately post-T-DXd, by HER2 status of the disease …………………………………...…………………………………….…………………....……………... 2
- **Supplementary Figure 2.** TTNT with regimens administered immediately post-T-DXd, by breast cancer subtype ………………………………………………………………………..…………………….……………………….. 3
- **Supplementary Figure 3.** TTNT with T-DXd according to HS-HER2 medians (HER2-negative patients only…... 4
- **Supplementary Figure 4.** TTNT (A) and OS (B) by presence of absence of ERBB2 hemizygous deletions among patients with HER2-negative metastatic breast cancer that received T-DXd and had available clinical NGS testing..... 5
- **Supplementary Figure 5**. Oncoprint of pre-T-DXd (A), post-T-DXd (B) plasma samples, and enrichment analysis (C)……………………………………………………………………………………………………. 6
- **Supplementary Table 1.** Dosing, dose reductions and toxicities experienced by patients receiving T-DXd in RELIEVE…………………..…………………..……………………………………………………………… 7
- **Supplementary Table 2.** Demographics of patients included in the HS-HER2 analysis………………………….. 9
- **Supplementary Table 3.** TTNT with T-DXd according to pre-treatment HS-HER2 levels…………….……….. 11
- **Supplementary Table 4.** OS with T-DXd according to pre-treatment HS-HER2 levels ……….……….……...... 12
- **Supplementary Table 5.** Demographics of patients included in the RPPA analysis …………………………….. 13
- **Supplementary Table 6.** TTNT with T-DXd according to pre-treatment HER2 RPPA levels ………………….. 15
- **Supplementary Table 7.** OS with T-DXd according to pre-treatment HER2 RPPA levels……..........................… 16
- **Supplementary Table 8.** TTNT with T-DXd according to pre-treatment phosphoHER2 Y1248 RPPA levels…………………………………………………………………...……………………...…………….. 17
- **Supplementary Table 9.** OS with T-DXd according to pre-treatment phosphoHER2 Y1248 RPPA levels……………………………………………………………………………………………………..... 18
- **Supplementary Table 10.** TTNT with T-DXd according to pre-treatment SLFN11 RPPA levels…………...….. 19
- **Supplementary Table 11.** TTNT with T-DXd according to pre-treatment TOPO1 RPPA levels……………….. 20
- **Supplementary Table 12.** Demographics of patients included in the HER2DX analysis……………………….… 21
- **Supplementary Table 13.** TTNT with T-DXd according to pre-treatment HER2 amplicon mRNA signature...... 23
- **Supplementary Table 14.** OS with T-DXd according to pre-treatment HER2 amplicon mRNA signature…..…. 24
- **Supplementary Table 15.** TTNT with T-DXd according to pre-treatment ERBB2 mRNA…………………….….. 25
- **Supplementary Table 16.** OS with T-DXd according to pre-treatment ERBB2 mRNA……………………..…. 26

SUPPLEMENTARY FIGURES

Supplementary Figure 1. TTNT with regimens administered immediately post-T-DXd, by HER2 status of the disease

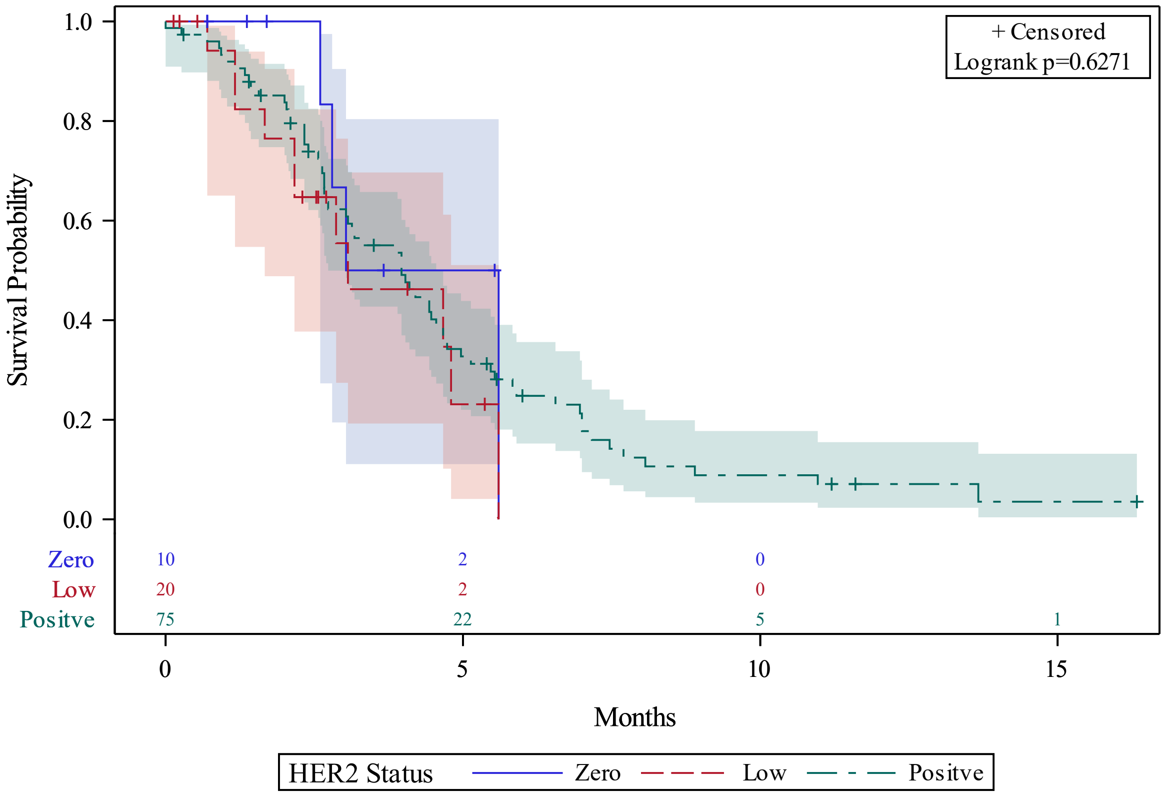

**HER2 Status**	**Median TTNT (95% CI)**	**p-value**
HER2-0	4.3 months (2.6 – 5.6)	0.6271
HER2-low	3.1 months (2.2 – 4.8)	
HER2-positive	4.0 months (2.7 – 4.6)	

Abbreviations: TTNT, time-to-next-treatment; T-DXd, trastuzumab deruxtecan; HER2, human epidermal growth factor receptor 2; CI, confidence interval

Supplementary Figure 2. TTNT with regimens administered immediately post-T-DXd, by breast cancer subtype

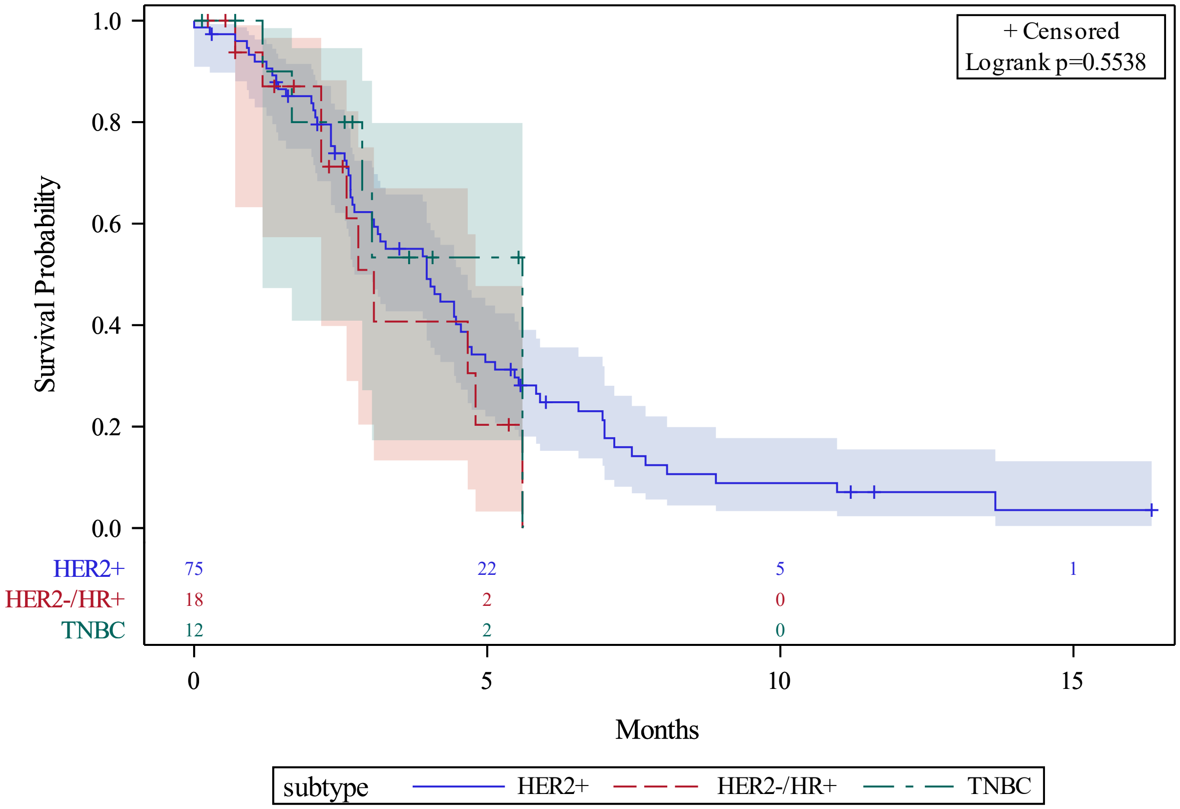

Breast Cancer Subtype	Median TTNT (95% CI)	p-value
HER2+	4.0 months (2.7 – 4.6)	0.5538
HR+/HER2-	3.1 months (2.2 – 4.8)	
TNBC	5.6 months (1.2 – 5.6)	

Abbreviations: TTNT, time-to-next-treatment; T-DXd, trastuzumab deruxtecan; HER2, human epidermal growth factor receptor 2; CI, confidence interval; HR, hormone receptor; TNBC, triple-negative breast cancer

Supplementary Figure 3. TTNT with T-DXd according to HS-HER2 medians (HER2-negative patients only)

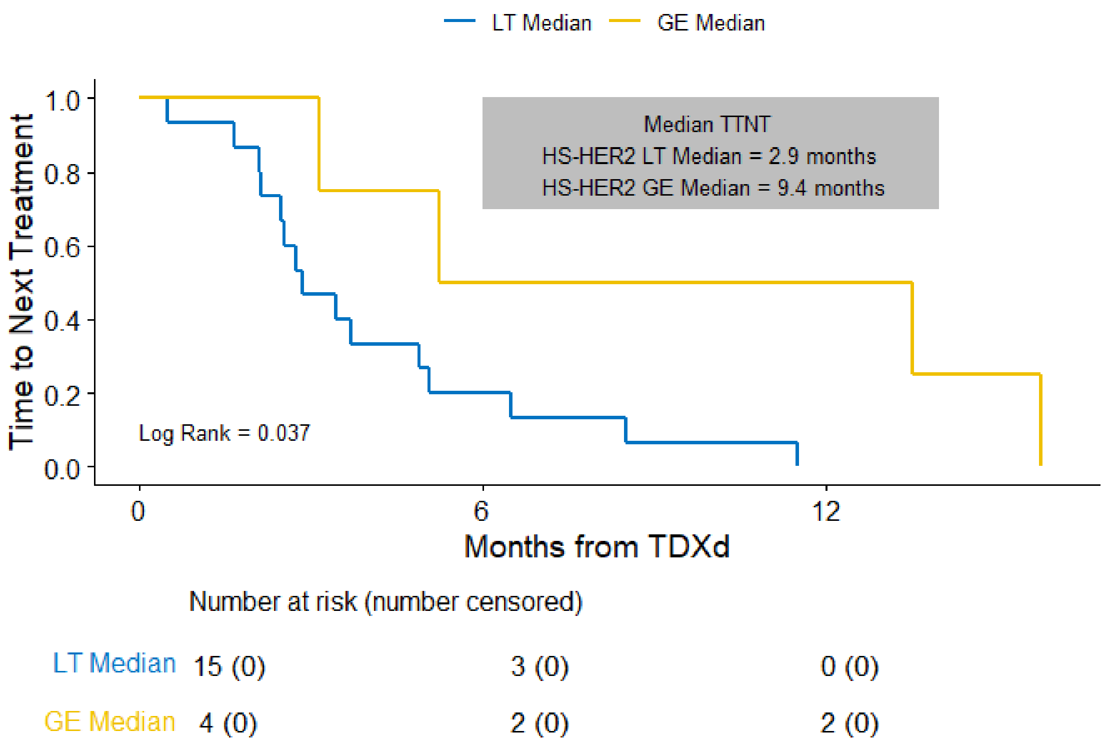

Abbreviations: TTNT, time-to-next-treatment; T-DXd, trastuzumab deruxtecan; HER2, human epidermal growth factor receptor 2

Supplementary Figure 4. TTNT (A) and OS (B) by presence of absence of ERBB2 hemizygous deletions among patients with HER2-negative metastatic breast cancer that received T-DXd and had available clinical NGS testing.

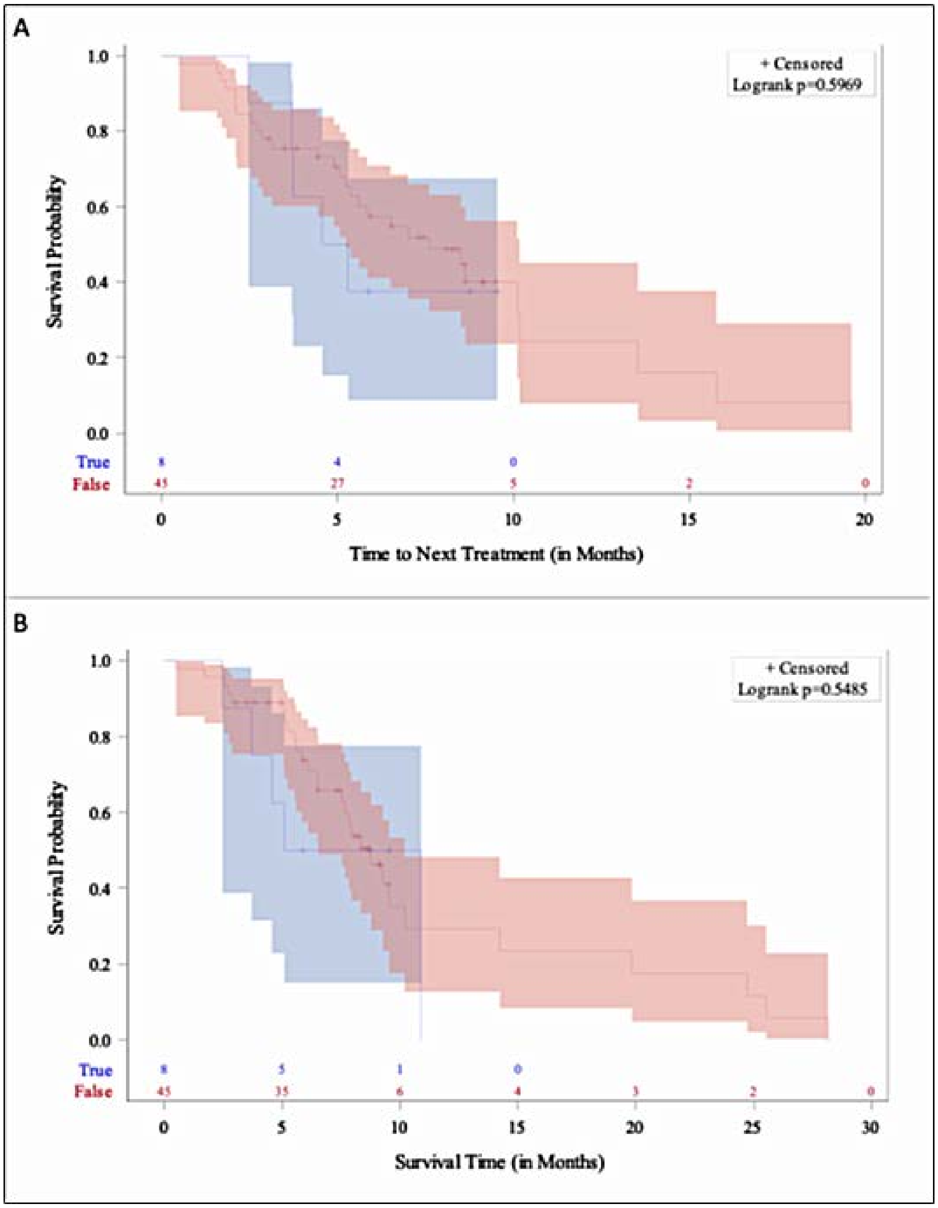

Abbreviations: TTNT, time-to-next-treatment; OS, overall survival; HER2, human epidermal growth factor receptor 2; T-DXd, trastuzumab deruxtecan; NGS, next-generation sequencing

Supplementary Figure 5. **Oncoprint of pre-T-DXd (A), post-T-DXd (B) plasma samples, and enrichment analysis (C).**

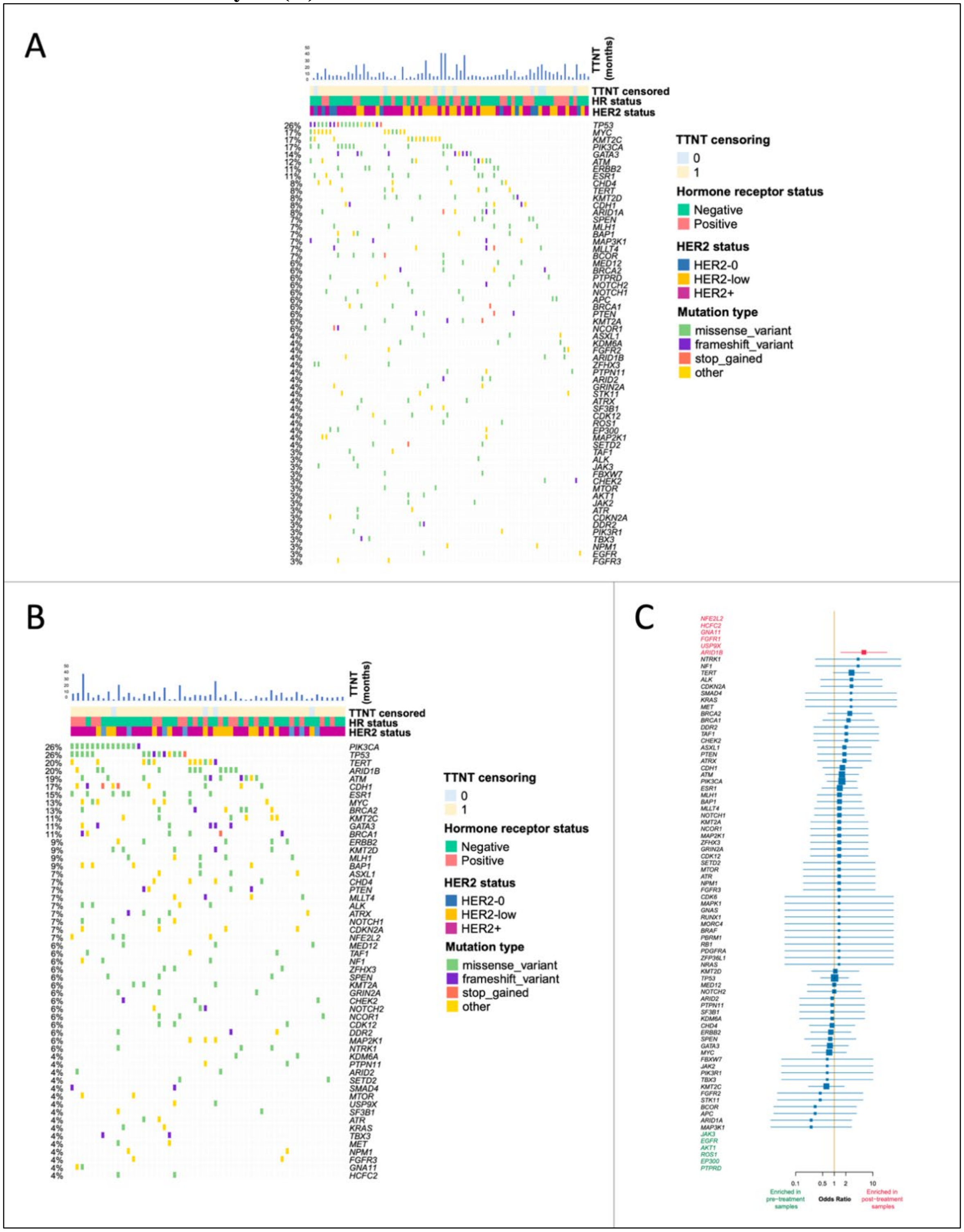

Abbreviations: T-DXd, trastuzumab deruxtecan; TTNT, time-to-next-treatment; HER2, human epidermal growth factor receptor 2

SUPPLEMENTARY TABLES

Supplementary Table 1. Dosing, dose reductions and toxicities experienced by patients receiving T-DXd in RELIEVE

	Total *n (%)*
**Starting Dose of T-DXd**	
3.2 mg/kg	10 (5.2%)
4.4 mg/kg	12 (6.3%)
5.4 mg/kg	150 (78.5%)
6.4 mg/kg	9 (4.7%)
Unknown	10 (5.2%)
**Required Dose Reduction during the course of treatment**	
Yes	61 (31.9%)
No	117 (61.3%)
Unknown	13 (6.8%)
**Levels of dose reduction required**	
1 level	41 (67.2%)
2 levels	19 (31.1%)
Unknown	1 (1.6%)
**Reason for Dose Reduction**	
Fatigue	28 (45.9%)
Hematological Toxicity	12 (19.7%)
Nausea/Vomiting	19 (31.1%)
Neuropathy	3 (4.9%)
Pulmonary Toxicity	6 (9.8%)
Cardiac Toxicity	1 (1.6%)
Abnormal LFTs	1 (1.6%)
Colitis, diarrhea	1 (1.6%)
Diarrhea, hepatic failure, acute kidney	1 (1.6%)
Mucositis	1 (1.6%)
Stomatitis, diarrhea	1 (1.6%)
Weakness, renal insufficiency	1 (1.6%)
Other	14 (23.0%)
**Cardiotoxicity Occurrence**	
Yes	5 (2.6%)
No	178 (93.2%)
Unknown, outside records not available	8 (4.2%)
**INCIDENCE AND CHARACTERIZATION OF ILD**	
**ILD Occurrence**	
Yes	22 (11.5%)
No	160 (83.8%)
Unknown	9 (4.7%)

	Total<br>*n (%)*
**Received steroid treatment for ILD**	
Yes	18 (81.8%)
No	4 (18.2%)
**Time from T-DXd Start to ILD onset** (in Months) [Median (Min, Max)]	8 (1, 51)
**Distribution of ILD Grade**	
Grade 1	12 (54.5%)
Grade 2	6 (27.3%)
Grade 3	3 (13.6%)
Grade 4	1 (4.5%)
**Type of ILD Diagnosis**	
Clinical and radiological	12 (54.5%)
Radiological only	10 (45.5%)
**Treatment received for ILD**	
Steroids	19 (86.4%)
Antibiotics	7 (31.8%)
Oxygen Therapy	3 (13.6%)
**ILD Resolved**	
Yes	14 (63.6%)
No	6 (27.3%)
Unknown	2 (9.1%)
**Time to ILD Resolution** (in Months) [Median (Min, Max)]	4 (1, 16)
**Prior History of pulmonary comorbidities among patients with ILD**	
Pneumonia	1 (4.5%)
Prior Asthma	1 (4.5%)
RSV Pneumonia	1 (4.5%)
Diagnosed with COPD on pulmonary consult	1 (4.5%)
**Smoking Status of patients with ILD**	
Current/Former Smoker	7 (31.8%)
Not Smoker	13 (59.1%)
Unknown	2 (9.1%)
**Pulmonary Consultation**	
Yes (multiple)	10 (45.5%)
Yes (once)	6 (27.3%)
No	4 (18.2%)
Unknown	2 (9.1%)

Abbreviations: T-DXd, trastuzumab deruxtecan; LFTs, liver function tests; ILD, interstitial lung disease; RSV, respiratory syncytial virus; COPD, chronic obstructive pulmonary disease

Supplementary Table 2. Demographics of patients included in the HS-HER2 analysis

	HER2-Positive (N=32)	HER2-Negative (N=19)	Total (N=51)
**Age at metastatic diagnosis**			
Median	49.2	53.1	51.4
Range	30.3 - 70.8	25.4 - 67.4	25.4 - 70.8
**Sex**			
Female	32 (100.0%)	19 (100.0%)	51 (100.0%)
**Time from metastatic diagnosis to initiation of T-DXd (Months)**			
Median	42.0	32.0	34.0
Range	1.0 - 137.0	2.0 - 155.0	1.0 - 155.0
**Number of metastatic sites at start of T-DXd**			
Median	3.0	3.0	3.0
Range	1.0 - 6.0	1.0 - 5.0	1.0 - 6.0
**HR Status at T-DXd**			
Positive	14 (43.8%)	9 (47.4%)	23 (45.1%)
Negative	18 (56.2%)	10 (52.6%)	28 (54.9%)
**HER2 status for primary tumor**			
HER2 Positive	24 (75.0%)	0 (0.0%)	24 (47.1%)
HER2 Negative	5 (15.6%)	18 (94.7%)	23 (45.1%)
Unknown	3 (9.4%)	1 (5.3%)	4 (7.8%)
**HER2 status for metastatic tumor**			
HER2 Positive	22 (68.6%)	0 (0.0%)	22 (43.1%)
HER2 Negative	4 (12.5%)	18 (94.7%)	22 (43.1%)
Unknown	6 (18.8%)	1 (5.3%)	7 (13.7%)
**HER2 Status for metastatic tumor (primary fallback)**			
Positive	27 (84.4%)	0 (0.0%)	27 (52.9%)
Negative	5 (15.6%)	19 (100.0%)	24 (47.1%)
**Prior lines of treatment in the advanced setting**			
Median	5.0	4.0	5.0
Range	1.0 - 16.0	0.0 - 10.0	0.0 - 16.0
**Prior lines of chemotherapy**			
Median	2.5	2.0	2.0
Range	0.0 - 9.0	0.0 - 6.0	0.0 - 9.0
**Prior lines of endocrine therapy**			
Median	0.0	1.0	0.0
Range	0.0 - 3.0	0.0 - 3.0	0.0 - 3.0
N/A (Always HR-)	14	3	17

	HER2-Positive (N=32)	HER2-Negative (N=19)	Total (N=51)
**Receipt of Sacituzumab Govitecan**			
Yes, before T-DXd	6 (18.8%)	12 (63.2%)	18 (35.3%)
Yes, after T-DXd	3 (9.4%)	4 (21.1%)	7 (13.7%)
No	23 (71.9%)	3 (15.8%)	26 (51.0%)
**HS-HER2 tumor type**			
Both	10 (31.2%)	8 (42.1%)	18 (35.3%)
Primary only	13 (40.6%)	7 (36.8%)	20 (39.2%)
Metastasis only	9 (28.1%)	4 (21.1%)	13 (25.5%)

Abbreviations: HER2, human epidermal growth factor receptor 2; MetDX, metastasis at the time of diagnosis; T-DXd, trastuzumab deruxtecan; HR, hormone receptor; T-DXD, trastuzumab deruxtecan

Supplementary Table 3. TTNT with T-DXd according to pre-treatment HS-HER2 levels

TTNT (all patients)						
	**Group**	**Median TTNT (month)**	**95% CI**	**HR**	**95% CI**	**p-value**
**Continuous (amol/mm2)**	1 unit increase	-	-	0.95	0.93-0.97	<0.001
	5 unit increase	-	-	0.77	0.68-0.86	<0.001
**Median**						(<0.001)
	< median (ref)	3.70	2.73-5.37			
	>= median	7.68	5.23-15.77	0.30	0.16-0.57	
**Quartile**						(<0.001)
	<=25% (ref)	3.43	2.53-NA			
	>25%-50%	5.07	2.10-NA	0.58	0.26-1.28	0.18
	>50%-75%	6.98	4.37-NA	0.26	0.11-0.64	0.003
	>75%	11.67	5.83-NA	0.16	0.06-0.40	<0.001
**TTNT (only HER2-positive)**						
	**Group**	**Median TTNT (month)**	**95% CI**	**HR**	**95% CI**	**p-value**
**Continuous (amol/mm2)**	1 unit increase	-	-	0.96	0.93-0.98	0.003
	5 unit increase	-	-	0.80	0.69-0.93	0.003
**Median**						(0.012)
	< median (ref)	5.03	3.27-NA			
	>= median	7.68	5.83-22.70	0.36	0.16-0.83	
**TTNT (only HER2-negative)**						
	**Group**	**Median TTNT (month)**	**95% CI**	**HR**	**95% CI**	**p-value**
**Continuous (amol/mm2)**	1 unit increase	-	-	0.93	0.87-0.98	0.011
	5 unit increase	-	-	0.68	0.51-0.92	0.011
**Median**						(0.037)
	< median (ref)	2.87	2.47-6.5			
	>= median	9.38	3.13-NA	0.23	0.05-1.03	

All of the p-values in blue and in parenthesis are from log-rank test and all p-values not in parenthesis are from wald test.

Abbreviations: TTNT, time-to-next-treatment; T-DXd, trastuzumab deruxtecan; HER2, human epidermal growth factor receptor 2; CI, confidence interval; HR, hormone receptor

Supplementary Table 4. OS with T-DXd according to pre-treatment HS-HER2 levels

OS (all patients)

	**Group**	**Median OS (month)**	**95% CI**	**HR**	**95% CI**	**p-value**
**Continuous (amol/mm2)**	1 unit increase	-	-	0.95	0.93-0.98	< 0.001
	5 unit increase	-	-	0.79	0.69-0.90	< 0.001
**Median**						(0.003)
	< median (ref)	7.8	4.67-16.2			
	>= median	20.2	13.07-NA	0.37	0.19-0.72	
**Quartile**						(0.001)
	<=25% (ref)	5.03	2.87-NA			
	>25%-50%	10.50	5.07-NA	0.45	0.19-1.09	0.079
	>50%-75%	20.17	9.53-NA	0.26	0.10-0.69	0.007
	>75%	27.23	13.07-NA	0.17	0.06-0.46	< 0.001
**OS (HER2-positive only)**						
	**Group**	**Median OS (month)**	**95% CI**	**HR**	**95% CI**	**p-value**
**Continuous (amol/mm2)**	1 unit increase	-	-	0.95	0.92-0.99	0.006
	5 unit increase	-	-	0.79	0.67-0.93	0.006
**Median**						(0.031)
	< median (ref)	12.83	5.03-NA			
	>= median	23.13	16.63-NA	0.39	0.16-0.94	
**OS (HER2-negative only)**						
	**Group**	**Median OS (month)**	**95% CI**	**HR**	**95% CI**	**p-value**
**Continuous (amol/mm2)**	1 unit increase	-	-	0.97	0.92-1.02	0.18
	5 unit increase	-	-	0.85	0.67-1.08	0.18
**Median**						(0.33)
	< median (ref)	5.07	2.73-NA			
	>= median	11.88	5.23-NA	0.53	0.15-1.91	

All of the p-values in blue and in parenthesis are from log-rank test and all p-values not in parenthesis are from wald test.

Abbreviations: OS, overall survival; T-DXd, trastuzumab deruxtecan; HER2, human epidermal growth factor receptor 2; CI, confidence interval; HR, hormone receptor

Supplementary Table 5. Demographics of patients included in the RPPA analysis

	HER2-Positive (N=24)	HER2-Negative (N=14)	Total (N=38)
**Age at metastatic diagnosis**			
Median	51.9	54.3	53.1
Range	30.3 - 70.8	33.1 - 65.3	30.3 - 70.8
**Gender**			
Female	24 (100.0%)	14 (100.0%)	38 (100.0%)
**Time from metastatic diagnosis to initiation of T-DXd (Months)**			
Median	59.0	35.5	37.0
Range	1.0 - 125.0	2.0 - 110.0	1.0 - 125.0
**Number of metastatic sites at start of T-DXd**			
Median	3.0	3.0	3.0
Range	1.0 - 6.0	1.0 - 5.0	1.0 - 6.0
**HR Status at T-DXd**			
Positive	9 (37.5%)	8 (57.1%)	17 (44.7%)
Negative	15 (62.5%)	6 (42.9%)	21 (55.3%)
**HER2 status for primary tumor**			
HER2 Positive	17 (81.0%)	0 (0.0%)	17 (50.0%)
HER2 Negative	4 (19.0%)	13 (100.0%)	17 (50.0%)
Unknown	3	1	4
**HER2 status for metastatic tumor**			
HER2 Positive	17 (70.8%)	0 (0.0%)	17 (44.7%)
HER2 Negative	2 (8.3%)	13 (92.9%)	15 (39.5%)
Unknown	5 (20.8%)	1 (7.1%)	6 (15.8%)
**HER2 status for metastatic tumor (primary fallback)**			
HER2 Positive	22 (91.7%)	0 (0.0%)	22 (57.9%)
HER2 Negative	2 (8.3%)	14 (100.0%)	16 (42.1%)
**Prior lines of treatment in the advanced setting**			
Median	5.0	4.0	5.0
Range	1.0 - 16.0	0.0 - 9.0	0.0 - 16.0
**Prior lines of chemotherapy**			
Median	3.0	2.0	2.0
Range	0.0 - 9.0	0.0 - 5.0	0.0 - 9.0
**Prior lines of endocrine therapy**			

	HER2-Positive (N=24)	HER2-Negative (N=14)	Total (N=38)
Median	0.0	1.0	0.5
Range	0.0 - 2.0	0.0 - 4.0	0.0 - 4.0
N/A (Always HR-)	11	1	12
**Receipt of sacituzumab govitecan**			
Yes, before T-DXd	4 (16.7%)	8 (57.1%)	12 (31.6%)
Yes, after T-DXd	2 (8.3%)	2 (14.3%)	4 (10.5%)
No	18 (75.0%)	4 (28.6%)	22 (57.9%)
**RPPA performed on**			
Primary only	13 (54.2%)	9 (64.3%)	22 (57.9%)
Metastasis only	8 (33.3%)	2 (14.3%)	10 (26.3%)
Both	3 (12.5%)	3 (21.4%)	6 (15.8%)

Abbreviations: RPPA, Reverse Phase Protein Array; HER2, human epidermal growth factor receptor 2; T-DXd, trastuzumab deruxtecan; HR, hormone receptor

Supplementary Table 6. TTNT with T-DXd according to pre-treatment HER2 RPPA levels

	Group	Median TTNT (month)	95% CI	HR	95% CI	p-value
**All patients (n=38)**						
**Continuous**	10-unit increase	-	-	0.95	0.90-1.01	0.083
**Median**						(0.004)
	< median (ref)	4.37	2.87-8.33			
	≥ median	8.00	5.97-23.13	0.37	0.18-0.74	
**Quartile**						(0.019)
	≤ 25% (ref)	4.03	2.87-NA			
	>25%-50%	5.83	2.13-NA	0.64	0.25-1.61	0.34
	>50%-75%	8.00	5.33-NA	0.28	0.10-0.74	0.011
	>75%	9.07	5.83-NA	0.30	0.12-0.75	0.010
**HER2+ at T-DXd (n=24)**						
**Continuous**	10-unit increase	-	-	0.97	0.91-1.03	0.28
**Median**						(0.009)
	< median (ref)	5.02	4.37-NA			
	≥ median	9.38	5.83-23.83	0.30	0.12-0.78	
**HER2- at T-DXd (n=14)**						
**Continuous**	10-unit increase	-	-	0.86	0.54-1.38	0.54
**Median**						(0.65)
	< median (ref)	3.13	2.53-NA			
	≥ median	6.50	4.90-NA	0.74	0.20-2.76	

All of the p-values in blue and in parenthesis are from log-rank test and all p-values not in parenthesis are from wald test.

Abbreviations: TTNT, time-to-next-treatment; T-DXd, trastuzumab deruxtecan; HR, hormone receptor; CI, confidence interval

Supplementary Table 7. OS with T-DXd according to pre-treatment HER2 RPPA levels

	Group	Median OS (month)	95% CI	HR	95% CI	p-value
**All patients (n=38)**						
**Continuous**	10 unit increase	-	-	0.89	0.82-0.98	0.015
**Median**						(< 0.001)
	< median (ref)	7.80	5.03-16.63			
	≥ median	27.23	20.17-NA	0.15	0.06-0.39	
**Quartile**						(< 0.001)
	≤ 25% (ref)	5.47	3.70-NA			
	>25%-50%	11.87	5.03-NA	0.68	0.26-1.75	0.42
	>50%-75%	27.23	20.17-NA	0.10	0.03-0.39	< 0.001
	>75%	25.07	18.63-NA	0.13	0.04-0.44	0.001
**HER2+ at T-DXd (n=24)**						
**Continuous**	10 unit increase	-	-	0.92	0.84-1.01	0.084
**Median**						(<0.001)
	< median (ref)	10.95	5.03-NA			
	≥ median	27.23	20.17-NA	0.16	0.05-0.53	
**HER2- at T-DXd (n=14)**						
**Continuous**	10 unit increase	-	-	0.66	0.36-1.23	0.19
**Median**						(0.14)
	< median (ref)	7.80	3.70-NA			
	≥ median	10.50	10.50-NA	0.24	0.03-1.89	

All of the p-values in blue and in parenthesis are from log-rank test and all p-values not in parenthesis are from wald test.

Abbreviations: OS, overall survival; T-DXd, trastuzumab deruxtecan; HER2, human epidermal growth factor receptor 2; CI, confidence interval; HR, hormone receptor

Supplementary Table 8. TTNT with T-DXd according to pre-treatment phosphoHER2 Y1248 RPPA levels

	Group	Median TTNT (month)	95% CI	HR	95% CI	p-value
**All patients (n=38)**						
**Continuous**	10 unit increase	-	-	0.89	0.78-1.02	0.087
**Median**						(0.069)
	< median (ref)	5.23	3.70-11.67			
	≥ median	7.03	5.33-22.70	0.54	0.27-1.06	
**Quartile**						(0.26)
	≤ 25% (ref)	5.23	4.37-NA			
	>25%-50%	4.35	2.53-NA	0.79	0.31-2.00	0.61
	>50%-75%	7.03	4.90-NA	0.58	0.23-1.42	0.23
	>75%	6.92	5.00-NA	0.40	0.16-1.04	0.061
**HER2+ at T-DXd (n=24)**						
**Continuous**	10 unit increase	-	-	0.92	0.80-1.06	0.24
**Median**						(0.14)
	< median (ref)	5.60	4.37-NA			
	≥ median	7.52	5.83-25.07	0.53	0.22-1.25	
**HER2- at T-DXd (n=14)**						
**Continuous**	10 unit increase	-	-	0.84	0.37-1.90	0.67
**Median**						(0.54)
	< median (ref)	3.70	2.53-NA			
	≥ median	4.90	3.13-NA	0.69	0.21-2.26	

All of the p-values in blue and in parenthesis are from log-rank test and all p-values not in parenthesis are from wald test.

Abbreviations: TTNT, time-to-next-treatment; T-DXd, trastuzumab deruxtecan; HER2, human epidermal growth factor receptor 2; RPPA, Reverse Phase Protein Array; CI, confidence interval; HR, hormone receptor

Supplementary Table 9. OS with T-DXd according to pre-treatment phosphoHER2 Y1248 RPPA levels

	Group	Median OS (month)	95% CI	HR	95% CI	p-value
			**All patients (n=38)**			
**Continuous**	10 unit increase	-	-	0.80	0.66-0.96	0.017
**Median**						(0.002)
	< median (ref)	9.53	5.03-19.23			
	≥ median	25.07	18.63-NA	0.30	0.13-0.67	
**Quartile**						(0.014)
	≤ 25%	5.70	5.03-NA			
	>25%-50%	13.08	2.87-NA	0.74	0.27-2.00	0.55
	>50%-75%	18.63	10.50-NA	0.34	0.11-0.99	0.048
	>75%	35.63	20.17-NA	0.19	0.06-0.61	0.005
			**HER2+ at T-DXd (n=24)**			
**Continuous**	10 unit increase	-	-	0.83	0.68-1.01	0.060
**Median**						(0.002)
	< median (ref)	14.63	5.03-NA			
	≥ median	35.63	20.17-NA	0.22	0.08-0.62	
			**HER2- at T-DXd (n=14)**			
**Continuous**	10 unit increase	-	-	0.77	0.31-1.95	0.58
**Median**						(0.44)
	< median (ref)	5.23	2.87-NA			
	≥ median	10.50	9.53-NA	0.61	0.18-2.14	

All of the p-values in blue and in parenthesis are from log-rank test and all p-values not in parenthesis are from wald test.

Abbreviations: OS, overall survival; T-DXd, trastuzumab deruxtecan; HER2, human epidermal growth factor receptor 2; RPPA, Reverse Phase Protein Array; CI, confidence interval; HR, hormone receptor

Supplementary Table 10. TTNT with T-DXd according to pre-treatment SLFN11 RPPA levels

	Group	Median TTNT (month)	95% CI	HR	95% CI	p-value
**All patients (n=38)**						
**Continuous**	10 unit increase	-	-	1.00	0.97-1.03	0.84
**Median**						(0.99)
	< median (ref)	7.03	5.23-13.53			
	≥ median	5.33	4.37-13.27	1.00	0.51-1.96	
**Quartile**						(0.58)
	≤ 25% (ref)	9.92	5.23-NA			
	>25%-50%	5.37	3.20-NA	1.58	0.64-3.94	0.32
	>50%-75%	5.00	4.37-NA	1.57	0.63-3.92	0.34
	>75%	5.90	2.10-NA	0.96	0.37-2.48	0.94
**HER2+ at T-DXd (n=24)**						
**Continuous**	10 unit increase	-	-	1.00	0.96-1.04	0.98
**Median**						(0.75)
	< median (ref)	7.52	5.37-NA			
	≥ median	5.90	5.00-25.07	0.87	0.37-2.06	
**HER2- at T-DXd (n=14)**						
**Continuous**	10 unit increase	-	-	1.08	1.00-1.17	0.048
**Median**						(0.09)
	< median (ref)	5.23	2.87-NA			
	≥ median	3.13	2.47-NA	2.85	0.81-10.07	

All of the p-values in blue and in parenthesis are from log-rank test and all p-values not in parenthesis are from wald test.

Abbreviations: TTNT, time-to-next-treatment; T-DXd, trastuzumab deruxtecan; RPPA, Reverse Phase Protein Array; CI, confidence interval; HR, hormone receptor; HER2, human epidermal growth factor receptor 2

Supplementary Table 11. TTNT with T-DXd according to pre-treatment TOPO1 RPPA levels

	Group	Median TTNT (month)	95% CI	HR	95% CI	p-value
**All patients (n=38)**						
**Continuous**	10 unit increase	-	-	1.01	0.95-1.08	0.73
**Median**						(0.96)
	< median (ref)	5.97	4.90-13.53			
	≥ median	5.37	3.20-13.27	0.98	0.51-1.90	
**Quartile**						(0.79)
	≤ 25% (ref)	9.00	5.23-NA			
	>25%-50%	4.90	4.67-NA	1.54	0.62-3.86	0.36
	>50%-75%	5.37	2.53-NA	1.06	0.41-2.72	0.90
	>75%	5.58	3.20-NA	1.29	0.53-3.13	0.58
**HER2+ at T-DXd (n=24)**						
**Continuous**	10 unit increase	-	-	1.01	0.93-1.09	0.82
**Median**						(0.50)
	< median (ref)	5.97	4.70-NA			
	≥ median	7.37	5.33-NA	0.75	0.32-1.74	
**HER2- at T-DXd (n=14)**						
**Continuous**	10 unit increase	-	-	1.17	0.97-1.40	0.093
**Median**						(0.036)
	< median (ref)	5.87	4.90-NA			
	≥ median	2.70	2.47-NA	3.49	1.02-11.96	

All of the p-values in blue and in parenthesis are from log-rank test and all p-values not in parenthesis are from wald test.

Abbreviations: TTNT, time-to-next-treatment; T-DXd, trastuzumab deruxtecan; RPPA, Reverse Phase Protein Array; CI, confidence interval; HR, hormone receptor; HER2, human epidermal growth factor receptor 2

Supplementary Table 12. Demographics of patients included in the HER2DX analysis

	HER2-Positive (N=25)	HER2-Negative (N=16)	Total (N=41)
**Age at metastatic diagnosis**			
Median	50.6	54.3	53.1
Range	30.3 - 70.8	33.9 - 65.3	30.3 - 70.8
**Gender**			
Female	25 (100.0%)	16 (100.0%)	41 (100.0%)
**Time from metastatic diagnosis to initiation of T-DXd (Months)**			
Median	50.0	27.5	34.0
Range	1.0 - 119.0	2.0 - 110.0	1.0 - 119.0
**Number of metastatic sites at start of T-DXd**			
Median	3.0	3.0	3.0
Range	1.0 - 6.0	1.0 - 5.0	1.0 - 6.0
**HR Status at T-DXd**			
Positive	10 (40.0%)	9 (56.2%)	19 (46.3%)
Negative	15 (60.0%)	7 (43.8%)	22 (53.7%)
**HER2 status for primary tumor**			
HER2 Positive	18 (72.0%)	0 (0.0%)	18 (43.9%)
HER2 Negative	4 (16.0%)	14 (87.5%)	18 (43.9%)
Unknown	3 (12.0%)	2 (12.5%)	5 (12.2%)
**HER2 status for metastatic tumor**			
HER2 Positive	18 (72.0%)	0 (0.0%)	18 (43.9%)
HER2 Negative	3 (12.0%)	15 (93.8%)	18 (43.9%)
Unknown	4 (16.0%)	1 (6.2%)	5 (12.2%)
**HER2 status for metastatic tumor (primary fallback)**			
HER2 Positive	22 (88.0%)	0 (0.0%)	22 (53.7%)
HER2 Negative	3 (12.0%)	16 (100.0%)	19 (46.3%)
**Prior lines of treatment in the advanced setting**			
Median	5.0	4.0	5.0
Range	1.0 - 16.0	0.0 - 9.0	0.0 - 16.0
**Prior lines of chemotherapy**			
Median	2.0	1.5	2.0
Range	0.0 - 9.0	0.0 - 5.0	0.0 - 9.0
**Prior lines of endocrine therapy**			
Median	0.0	1.0	1.0
Range	0.0 - 2.0	0.0 - 4.0	0.0 - 4.0
N/A (Always HR-)	13	1	14

	HER2-Positive (N=25)	HER2-Negative (N=16)	Total (N=41)
**Receipt of sacituzumab govitecan**			
Yes, before T-DXd	5 (20.0%)	8 (50.0%)	13 (31.7%)
Yes, after T-DXd	3 (12.0%)	4 (25.0%)	7 (17.1%)
No	17 (68.0%)	4 (25.0%)	21 (51.2%)
**HER2DX performed on**			
Primary only	14 (56.0%)	8 (50.0%)	22 (53.7%)
Metastasis only	7 (28.0%)	4 (25.0%)	11 (26.8%)
Both	4 (16.0%)	4 (25.0%)	8 (19.5%)

Abbreviations: HER2, human epidermal growth factor receptor 2; T-DXd, trastuzumab deruxtecan; HR, hormone receptor

Supplementary Table 13. TTNT with T-DXd according to pre-treatment HER2 amplicon mRNA signature

TTNT						
	**Group**	**Median TTNT (month)**	**95% CI**	**HR**	**95% CI**	**p-value**
**All patients (n=41)**						
**Continuous**	1 unit increase	-	-	0.70	0.56-0.87	0.001
**Group**						(0.019)
	Low (ref)	4.52	3.13-7.23			
	Medium	5.33	4.7-NA	0.71	0.33-1.55	0.39
	High	12.02	7.37-NA	0.23	0.08-0.69	0.009
**HER2+ at T-DXd (n=25)**						
**Continuous**	1 unit increase	-	-	0.63	0.47-0.86	0.004
**Group**						(0.012)
	Low (ref)	4.70	3.27-NA			
	Medium	5.33	4.7-NA	0.57	0.22-1.5	0.25
	High	12.02	7.37-NA	0.17	0.05-0.59	0.006
**HER2- at T-DXd (n=16)**						
**Continuous**	1 unit increase	-	-	0.45	0.21-0.94	0.033
**Group***						(-)
	Low (ref)	3.57	2.73-11.5			
	Medium			-	-	-
	High			-	-	-

All of the p-values in blue and in parenthesis are from log-rank test and all p-values not in parenthesis are from wald test.
*: there are no patients in medium and high groups

Abbreviations: TTNT, time-to-next-treatment; T-DXd, trastuzumab deruxtecan; HER2, human epidermal growth factor receptor 2; CI, confidence interval; HR, hormone receptor

Supplementary Table 14. OS with T-DXd according to pre-treatment HER2 amplicon mRNA signature

	Group	Median OS (month)	95% CI	HR	95% CI	p-value
**All patients (n=41)**						
**Continuous**	1 unit increase	-	-	0.65	0.5-0.84	0.001
**Group**						(0.009)
	Low (ref)	9.53	5.7-16.2			
	Medium	18.63	4.7-NA	0.36	0.13-1.00	0.050
	High	37.20	25.07-NA	0.15	0.04-0.6	0.007
**HER2+ at T-DXd (n=25)**						
**Continuous**	1 unit increase	-	-	0.58	0.40-0.83	0.003
**Group**						(0.008)
	Low (ref)	9.20	5.03-NA			
	Medium	18.63	4.7-NA	0.32	0.11-0.96	0.043
	High	37.20	25.07-NA	0.13	0.03-0.57	0.007
**HER2- at T-DXd (n=16)**						
**Continuous**	1 unit increase	-	-	0.52	0.24-1.15	0.108
**Group***						(-)
	Low (ref)	9.53	5.23-NA			
	Medium	-	-	-	-	-
	High	-	-	-	-	-

All of the p-values in blue and in parenthesis are from log-rank test and all p-values not in parenthesis are from wald test.
*: there are no patients in medium and high groups

Abbreviations: OS, overall survival; T-DXd, trastuzumab deruxtecan; HER2, human epidermal growth factor receptor 2; CI, confidence interval; HR, hormone receptor

Supplementary Table 15. TTNT with T-DXd according to pre-treatment ERBB2 mRNA

	Group	Median TTNT (month)	95% CI	HR	95% CI	p-value
**All patients (n=41)**						
**Continuous**	1 unit increase	-	-	0.76	0.64-0.91	0.002
**Group**						(0.002)
	Low (ref)	3.43	2.73-6.5			
	Medium	10.77	7.03-NA	0.26	0.08-0.79	0.018
	High	5.90	5.00-23.83	0.31	0.15-0.66	0.002
**HER2+ at T-DXd (n=25)**						
**Continuous**	1 unit increase	-	-	0.72	0.56-0.92	0.008
**Group**						(0.022)
	Low (ref)	4.67	2.10-NA			
	Medium	7.52	7.03-NA	0.31	0.06-1.54	0.15
	High	5.90	5.00-23.83	0.26	0.1-0.74	0.011
**HER2- at T-DXd (n=16)**						
**Continuous**	1 unit increase	-	-	0.44	0.22-0.9	0.024
**Group***						(0.016)
	Low (ref)	3.28	2.73-8.50			
	Medium	14.65	13.53-NA	0	0-Inf	> 0.99
	High	-	-	-	-	-

All of the p-values in blue and in parenthesis are from log-rank test and all p-values not in parenthesis are from wald test.
*: there are no patients in high group

Abbreviations: TTNT, time-to-next-treatment; T-DXd, trastuzumab deruxtecan; CI, confidence interval; HR, hormone receptor; HER2, human epidermal growth factor receptor 2

Supplementary Table 16. OS with T-DXd according to pre-treatment ERBB2 mRNA

	Group	Median OS (month)	95% CI	HR	95% CI	p-value
**All patients (n=41)**						
**Continuous**	1 unit increase	-	-	0.7	0.56-0.87	0.001
**Group**						(0.005)
	Low (ref)	9.53	5.03-14.4			
	Medium	20.17	14.23-NA	0.36	0.10-1.28	0.11
	High	25.07	13.07-NA	0.24	0.09-0.61	0.003
**HER2+ at T-DXd (n=25)**						
**Continuous**	1 unit increase	-	-	0.65	0.49-0.87	0.003
**Group**						(0.005)
	Low (ref)	7.13	4.67-NA			
	Medium	21.27	20.17-NA	0.26	0.05-1.48	0.13
	High	25.07	13.07-NA	0.16	0.05-0.55	0.003
**HER2- at T-DXd (n=16)**						
**Continuous**	1 unit increase	-	-	0.47	0.22-1.04	0.063
**Group***						(0.24)
	Low (ref)	9.53	5.23-NA			
	Medium	14.23	14.23-NA	0.3	0.04-2.44	0.26
	High	-	-	-	-	-

All of the p-values in blue and in parenthesis are from log-rank test and all p-values not in parenthesis are from wald test.
*: there are no patients in high group

Abbreviations: OS, overall survival; T-DXd, trastuzumab deruxtecan; CI, confidence interval; HR, hormone receptor; HER2, human epidermal growth factor receptor 2
